# TRPC1 promotes the genesis and progression of colorectal cancer via activating CaM-mediated PI3K/AKT signaling axis

**DOI:** 10.1038/s41389-021-00356-5

**Published:** 2021-10-12

**Authors:** Yang Sun, Chen Ye, Wen Tian, Wen Ye, Yuan-Yuan Gao, Ying-Da Feng, Hui-Nan Zhang, Guang-Yuan Ma, Shou-Jia Wang, Wei Cao, Xiao-Qiang Li

**Affiliations:** 1 Department of Pharmacology, School of Pharmacy, Fourth Military Medical University, Xi’an, Shaanxi 710032 China; 2 Key Laboratory of Gastrointestinal Pharmacology of Chinese Materia Medica of the State Administration of Traditional Chinese Medicine, Xi’an, Shaanxi 710032 China; 3 Department of Pharmacy, School of Chemistry & Pharmacy, Northwest A&F University, Yangling, Shaanxi 712100 China

**Keywords:** Ion channel signalling, Colorectal cancer, Oncogenes

## Abstract

Transient receptor potential canonical (TRPC) channels are the most prominent nonselective cation channels involved in various diseases. However, the function, clinical significance, and molecular mechanism of TRPCs in colorectal cancer (CRC) progression remain unclear. In this study, we identified that *TRPC1* was the major variant gene of the TRPC family in CRC patients. TRPC1 was upregulated in CRC tissues compared with adjacent normal tissues and high expression of TRPC1 was associated with more aggressive tumor progression and poor overall survival. TRPC1 knockdown inhibited cell proliferation, cell-cycle progression, invasion, and migration in vitro, as well as tumor growth in vivo; whereas TRPC1 overexpression promoted colorectal tumor growth and metastasis in vitro and in vivo. In addition, colorectal tumorigenesis was significantly attenuated in *Trpc1*^-/-^ mice. Mechanistically, TRPC1 could enhance the interaction between calmodulin (CaM) and the PI3K p85 subunit by directly binding to CaM, which further activated the PI3K/AKT and its downstream signaling molecules implicated in cell cycle progression and epithelial-mesenchymal transition. Silencing of CaM attenuated the oncogenic effects of TRPC1. Taken together, these results provide evidence that TRPC1 plays a pivotal oncogenic role in colorectal tumorigenesis and tumor progression by activating CaM-mediated PI3K/AKT signaling axis. Targeting TRPC1 represents a novel and specific approach for CRC treatment.

## Introduction

Colorectal cancer is one of the most common malignant tumors in the world [1]. Both the incidence and fatality rate of colorectal cancer are increasing rapidly and maintaining an upward trend in developed countries [1, 2]. The molecular mechanisms in colorectal cancer initiation and progression are complex and heterogeneous, involving chromosomal instability, microsatellite instability, and abnormal gene amplification, deletion or expression [3–5]. Alteration of multiple genes, such as oncogene or tumor suppressor gene, leading to disordered signal transduction, is one of the major mechanisms in colorectal cancer. Despite the high prevalence of gene expression change, their role in the pathogenesis of colorectal cancer remains poorly understood [6]. Thus, identification of novel driver genes may uncover oncogenic pathways underlying the initiation and progression of colorectal cancer and discover potential targets for CRC treatment.

TRPC channels are the first identified members in the TRP family, and constitute a group of calcium-permeable nonselective cation channels, which mediates the influx of Ca^2+^ and other cations into the cytosol of cells [7, 8]. Based on their amino acid sequences and functional similarities, the seven mammalian members of the TRPC subfamily fall into four subgroups: TRPC1, TRPC2, TRPC4/5, and TRPC3/6/7 that contribute to a broad spectrum of cellular functions and physiological roles [7, 9]. TRPCs have been shown to emerge as potential regulators in various physiological and pathological processes, such as myocardial hypertrophy [10], angiogenesis [11], and diabetic nephropathy [12]. In recent years, several TRPC members participated in diverse cell functions involved in tumor progression, such as cell proliferation, apoptosis, and migration [13, 14], and the p38-MAPK, JNK, and Ras/Raf1/ERK signaling are involved in these processes [15, 16]. However, investigations on the function, clinical significance, and molecular targets of the TRPC subfamily in CRC progression still remain limited.

In this study, we systematically uncovered the oncogenic role of TRPC1 on colorectal cancer cell functions and tumorigenicity in both primary CRC mice using *Trpc1* knockout (*Trpc1*^*-/-*^) and nude mice xenografted with gain or loss of TRPC1 human CRC cells. To explore the potential mechanisms, we performed STRING analysis and found that calmodulin (CaM) was closely related to it. CaM, a small intracellular calcium-binding protein, expressed in all eukaryotic cells and participated in signaling pathways that regulate many crucial processes such as growth, proliferation, and movement [17, 18]. CaM and Phosphatidylinositol 3-kinases (PI3K) are common components of several fundamental intracellular processes, and PI3K activity is enhanced by CaM association with the p85 subunit of PI3K [19]. Whether TRPCs can activate PI3K signaling through CaM is largely unknown. Here, we showed that TRPC1 directly interacts with CaM, which promotes molecular interactions between CaM and the PI3K p85 subunit, leading to activation of PI3K/AKT signaling cascade. Thus, TRPC1 plays a pivotal oncogenic role in CRC via activating CaM-mediated PI3K/AKT signaling axis.

## Results

### TRPC1 upregulation is correlated with tumor progression and poor prognosis of CRC patients

**Fig. 1:**
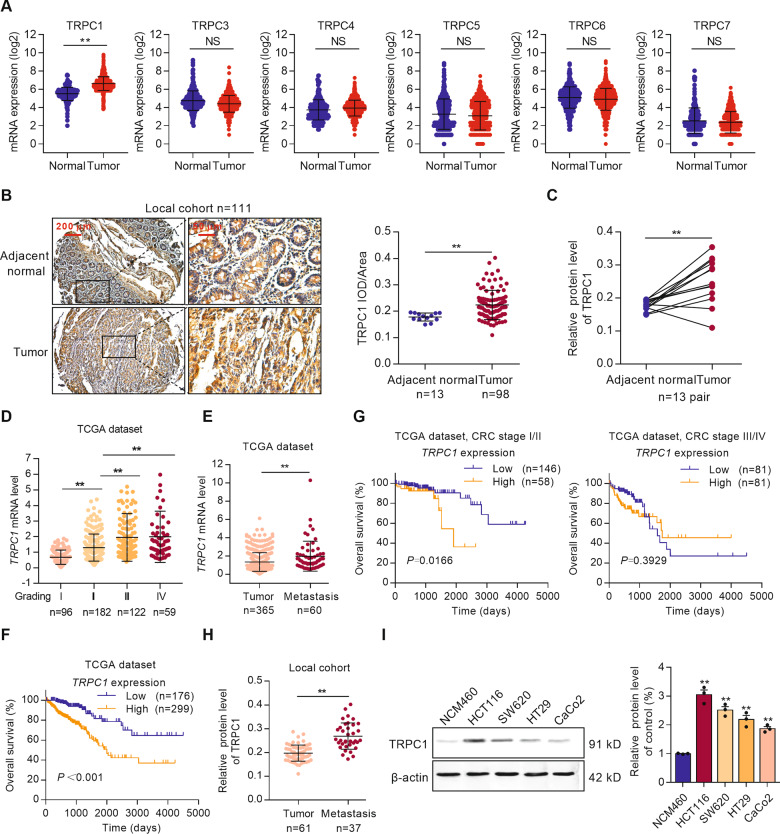
TRPC1 upregulation in CRC is positively correlated with tumor progression and poor prognosis of CRC patients. **A** Analyses of *TRPCs* mRNA expression in adjacent normal tissues (*n* = 287) and colorectal tumor tissues (*n* = 1994) in the GENT2 dataset. **B** Protein expression of TRPC1 in colorectal tumor tissues (*n* = 98) and adjacent normal tissues (*n* = 13) from the local cohort were assessed by IHC. **C** TRPC1 expression in 13 paired colorectal cancer and adjacent normal tissues from the local cohort were assessed by IHC. **D** Analyses of the *TRPC1* mRNA expression in different CRC tumor stage (*n* = 459) in the TCGA database. **E** Analyses of *TRPC1* mRNA levels in primary colorectal tumors (*n* = 365) and metastasis tumors (*n* = 60) in the TCGA database. **F** Kaplan–Meier survival analysis of overall survival according to the *TRPC1* mRNA expression in the TCGA database. High *TRPC1* expression predicts poor overall survival in colorectal cancer. **G** Kaplan–Meier survival analysis of overall survival according to the *TRPC1* mRNA expression in CRC patients with stage I/II or stage III/IV in the TCGA database. **H** Analyses of the TRPC1 protein expression in primary colorectal tumors (*n* = 61) and metastasis tumors (*n* = 37) in local cohort by IHC. **I** TRPC1 was frequently upregulated in CRC cell lines as determined by Western blot analysis (*n* = 3). **P < 0.01 vs. NCM460 group. **P < 0.01.

To identify the correlation between TRPCs and CRC, gene expression data were analyzed in the GENT2 database. The results showed that *TRPC1* mRNA expression was significantly higher in CRC tissues (*n* = 1994) compared with normal colorectal tissues (*n* = 287), while other members of the TRPC family did not show any significant change (Fig. 1A). Consistent with the increased *TRPC1* mRNA, TRPC1 protein expression also markedly increased 1.26-fold and 1.41-fold in tumor tissues compared with adjacent normal tissues in both individual CRC samples (*n* = 98) and additional 13 pairs of samples in the local cohort (Fig. 1B, C). In general, these data reveal that TRPC1 is aberrantly upregulated in CRC.

To evaluate the clinicopathologic features and prognostic significance of TRPC1, we further investigated the association between TRPC1 and the CRC stage in the TCGA database. There was a striking increase in the mRNA levels of *TRPC1* in the advanced stage (III/IV) compared with the early stage (I/II) (Fig. 1D), suggesting that *TRPC1* level increases during tumor progression. Consistently, the mRNA expression of *TRPC1* in the metastasis tumor tissues was significantly higher than the primary colorectal tumor tissues, which indicated that *TRPC1* upregulation was related to CRC deterioration (Fig. 1E). Furthermore, the Kaplan–Meier survival analysis indicated that CRC patients with higher levels of *TRPC1* had a worse outcome (*P* < 0.001, Fig. 1F), especially in early-stage (I/II) patients, but not in advanced-stage (III/IV) patients (Fig. 1G). The disease-free survival of CRC patients with high levels of *TRPC1* also decreased compared with those with low expression (*P* = 0.0397, Supplementary Fig. S1B). By contrast, the levels of *TRPC3, TRPC4, TRPC6* mRNA do not correlate with the overall survival of CRC patients (Supplementary Fig. S1C). Since TRPC1 was a major changed subtype of TRPC family, it was the focus of the subsequent study.

Further analysis of TRPC1 levels in primary and metastatic tumors of human CRC tissue microarray demonstrated that the TRPC1 protein expression was markedly enhanced in human metastatic CRC tissues (Fig. 1H). The protein levels of TRPC1 in four CRC cell lines (HCT116, HT29, SW620, and CaCo2) and human colonic epithelial cell NCM460 were also detected by Western blot. Similarly, the protein levels of TRPC1 were significantly increased in CRC cell lines and TRPC1 expression was related to the invasive capacity of CRC cells (Fig. 1I).

### TRPC1 knockdown suppresses cell proliferation and cell-cycle progression in vitro

**Fig. 2:**
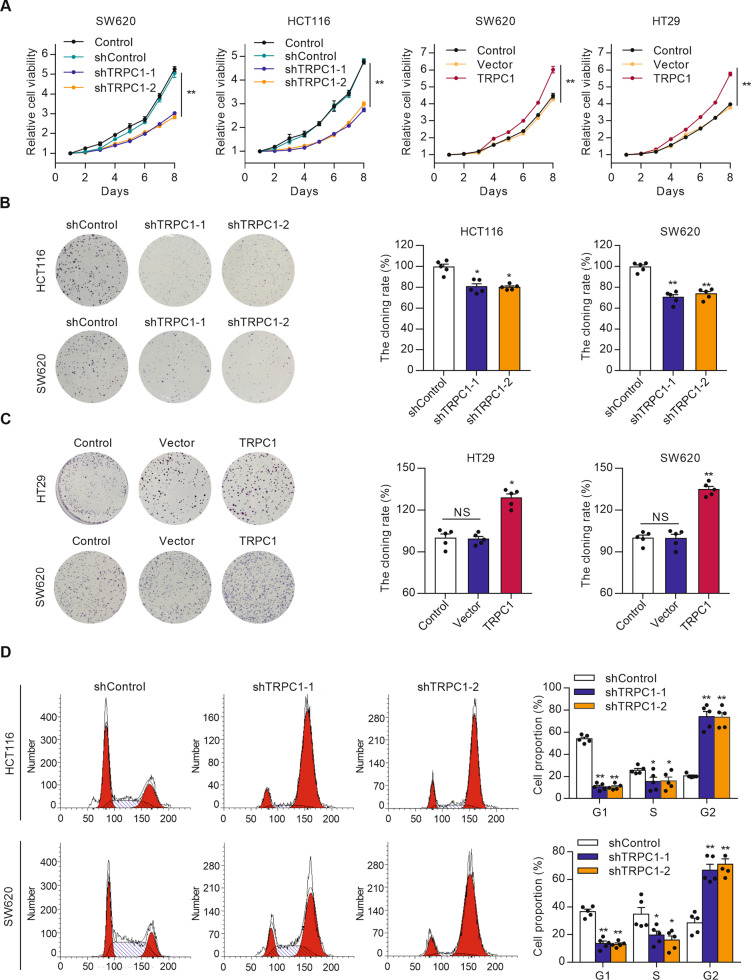
TRPC1 knockdown inhibits cell proliferation and cell-cycle progression in colorectal cancer cells. **A** MTT assay exhibited that forced expression of TRPC1 promoted cell growth, whereas cell growth was inhibited by TRPC1 knockdown (*n* = 5). **B** Knockdown of TRPC1 suppressed colony formation in the HCT116 and SW620 cells. Typical results are shown in the left panel along with the statistical analysis in the right panel (*n* = 5). **C** Forced expression of TRPC1 promoted colony formation in the HT29 and SW620 cells (*n* = 5). **D** The effects of TRPC1 knockdown on cell cycles in the HCT116 and SW620 cells (*n* = 5). Typical flow cytometric histograms (left panel) and the cell cycle distribution (right panel) were shown. *P < 0.05; **P < 0.01 vs. control.

To gain insight into the biological role of TRPC1 in CRC, TRPC1 stably overexpressing SW620 and HT29 cells, and TRPC1 stably knockdown HCT116 and SW620 cells were constructed by lentiviral shRNA vectors. Any successful silence or overexpression was identified by fluorescence imaging and Western blot analysis (Supplementary Fig. S2A, B). MTT assays revealed that depletion of TRPC1 caused evident compromised viability in both the HCT116 (55.4% of control at day 8) and SW620 (54.3% of control at day 8) cells. On the contrary, accessorial expression of TRPC1 significantly promoted cell viability both in the SW620 (149.7% of control at day 8) and HT29 (153.1% of control at day 8) cells (Fig. 2A). These results were further validated in colony formation assays (Fig. 2B, C). To investigate the possible mechanism by which TRPC1 promotes CRC cell proliferation, the cell cycle distribution was examined by flow cytometry. TRPC1 knockdown in the HCT116 and SW620 cells markedly decreased the G1 to S phase cell population, with a corresponding increase in the G2 phase population (Fig. 2D), indicating that TRPC1 knockdown causes cell cycle arrest in the G2 phase. These findings uncover that TRPC1 plays an oncogenic role in promoting cell growth and accelerating cell-cycle progression in CRC cells.

### TRPC1 motivates tumor growth and tumorigenesis in vivo

**Fig. 3:**
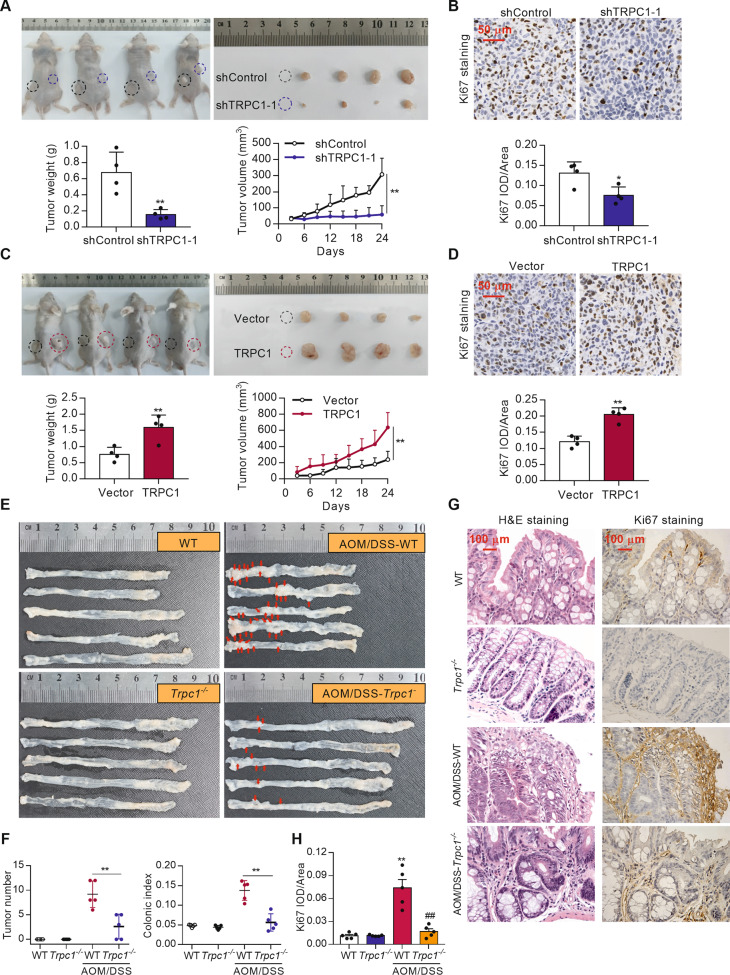
TRPC1 knockdown suppresses tumor genesis and growth in vivo. **A** TRPC1 knockdown inhibited the growth of xenotransplanted SW620 cells and decreased the tumor weight and volume in nude mice (*n* = 4). **B** Cell proliferation in tumor tissues of the nude mice xenotransplanted with TRPC1 knockdown or the control SW620 cells as evaluated by Ki67 staining. The representative tumor tissues are shown in the upper panel along with the quantitative analysis in the lower panel (*n* = 4). IOD integrated optical density. **C** Overexpression of TRPC1 promoted the growth of xenotransplanted SW620 cells and increased the tumor weight and volume in nude mice (*n* = 4). **D** Cell proliferation in tumor tissues of the nude mice xenotransplanted with TRPC1 overexpression or the vector SW620 cells as evaluated by Ki67 staining (*n* = 4). **E** The effect of *Trpc1* knockout on tumor genesis in AOM/DSS-induced CRC mice (*n* = 5). Dissection micrographs of colorectal tissues from each group. **F** Statistic analysis of tumor number and colonic index (colorectum weight / total body weight) in each group (*n* = 5). **G** Representative hematoxylin and eosin (H&E) stained or Ki67 stained colorectal tissues. **H** Statistic analysis of Ki67-positive index in each group. Ki67-positive index was quantified by counting the proportion of nuclear Ki67-positive cells (*n* = 5). **P < 0.01 vs. WT group; ^##^*P* < 0.01 vs. AOM/DSS-WT group.

To further confirm the growth-enhancing effect of TRPC1 in vivo, SW620 cells stably expressing sh-TRPC1 or sh-control, TRPC1 or vector were subcutaneously injected into both dorsal flanks of nude mice for xenotransplantation. The TRPC1 knockdown group demonstrated a smaller mean tumor volume and weight than the control cells and the inhibited rate was 77.2% (Fig. 3A). Moreover, the expression of Ki67, a marker of cell proliferation, in the SW620 xenografted tumors was examined by immunohistochemistry. TRPC1 depletion significantly suppressed Ki67 expression in these xenografts, confirming that TRPC1 knockdown inhibited the proliferation of CRC cells in vivo (Fig. 3B). Contrarily, TRPC1 overexpression markedly facilitated tumor growth, concomitant with increased cell proliferation (Fig. 3C, D). These data provide evidence that TRPC1 markedly accelerates the growth of xenografted colorectal tumors in vivo.

To further determine the tumorigenesis effect of TRPC1 in vivo, a classical AOM/DSS-induced method was used to induce colorectal carcinogenesis in *Trpc1*^-/-^ mice and WT mice. The specific experimental process is shown in Supplementary Fig. S3A. Similar to human CRC tissues, TRPC1 protein level was significantly higher in the AOM/DSS-induced colorectal tissues of WT mice than those in the control mice (Supplementary Fig. S3B). Compared with control mice, the tumor number and colonic index were significantly augmented to 9.2-fold and 2.9-fold, respectively, in the AOM/DSS-induced WT mice. Importantly, this augmentation was strikingly blunted to 2.6-fold and 1.2-fold, respectively, in the AOM/DSS-induced *Trpc1*^-/-^ mice (Fig. 3E, F). Consistently, there was significant receded cell proliferation in tumor tissues of the AOM/DSS-*Trpc1*^-/-^ mice compared with the AOM/DSS-WT group as analyzed by Ki67 staining (Fig. 3G, H). Collectively, these findings demonstrate that TRPC1 plays an important oncogenic role in motivating tumor growth and tumorigenesis in vivo.

### TRPC1 induces cell invasion and migration in vitro and tumor metastasis in vivo

**Fig. 4:**
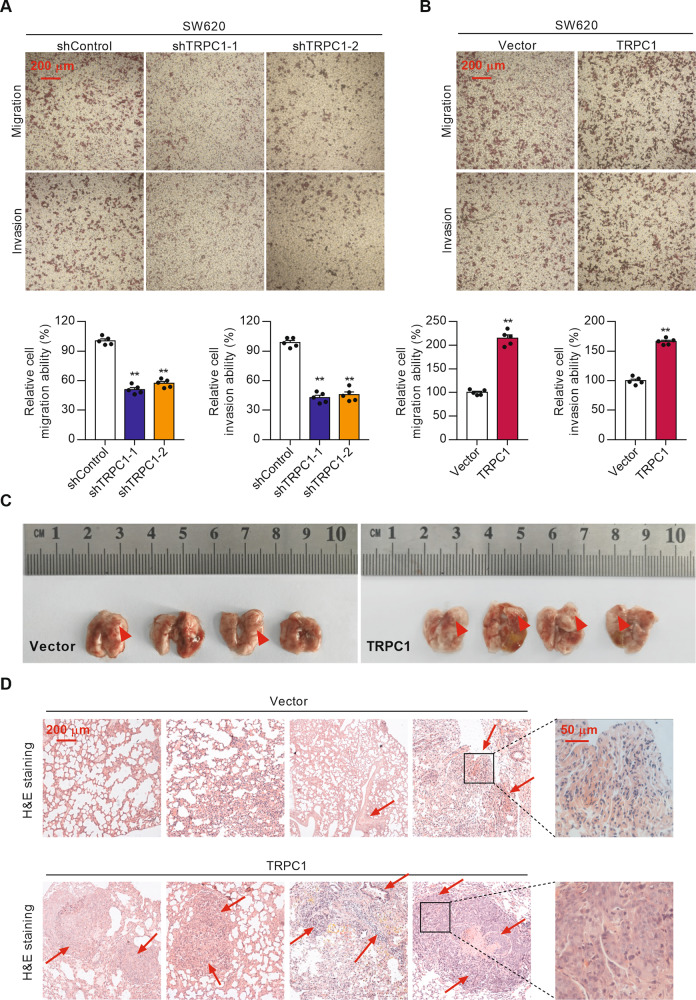
TRPC1 induces cell invasion and migration in vitro and tumor metastasis in vivo. **A** TRPC1 knockdown inhibited the invasive and migratory capabilities of SW620 cells. Typical results are shown in the upper panel along with the statistical analysis in the lower panel (*n* = 5). **B** Overexpression of TRPC1 facilitated the invasive and migratory capabilities of SW620 cells (*n* = 5). **C** Images of lung metastasis in nude mice inoculated with vector or TRPC1 overexpressed SW620 cells (*n* = 4). **D** Representative H&E stained lung metastases tissues of nude mice. Images were observed under 100× magnification. **P < 0.01.

To determine the effect of TRPC1 on cell migration and invasion, transwell assays were performed in CRC cells. The results showed that the suppression of TRPC1 resulted in a significantly diminished invasive and migratory potential in both the SW620 and HCT116 cells compared with control cells (Fig. 4A and Supplementary Fig. S4A). In contrast, a marked increase in cell invasion and migration ability was observed in the cells with elevated TRPC1 expression (Fig. 4B and Supplementary Fig. S4B). To further investigate its role on tumor metastasis in vivo, SW620 cells stably expressing TRPC1 or vector were inoculated into the tail vein of nude mice. Compared with the vector group, the overexpression of TRPC1 resulted in a greater lung metastasis burden (200% tumor incidence of control) (Fig. 4C). Moreover, H&E-stained lung sections revealed that TRPC1-overexpressed tumors showed extensive tumor tuberous tissues (Fig. 4D), which is consistent with the increased cell proliferation in metastases lung tissues (Supplementary Fig. S3C). Collectively, these data suggest that TRPC1 induces cell invasion and migration in vitro and tumor metastasis in vivo.

### TRPC1 modulates the expression of multiple biomarkers involved in cell cycle and EMT

**Fig. 5:**
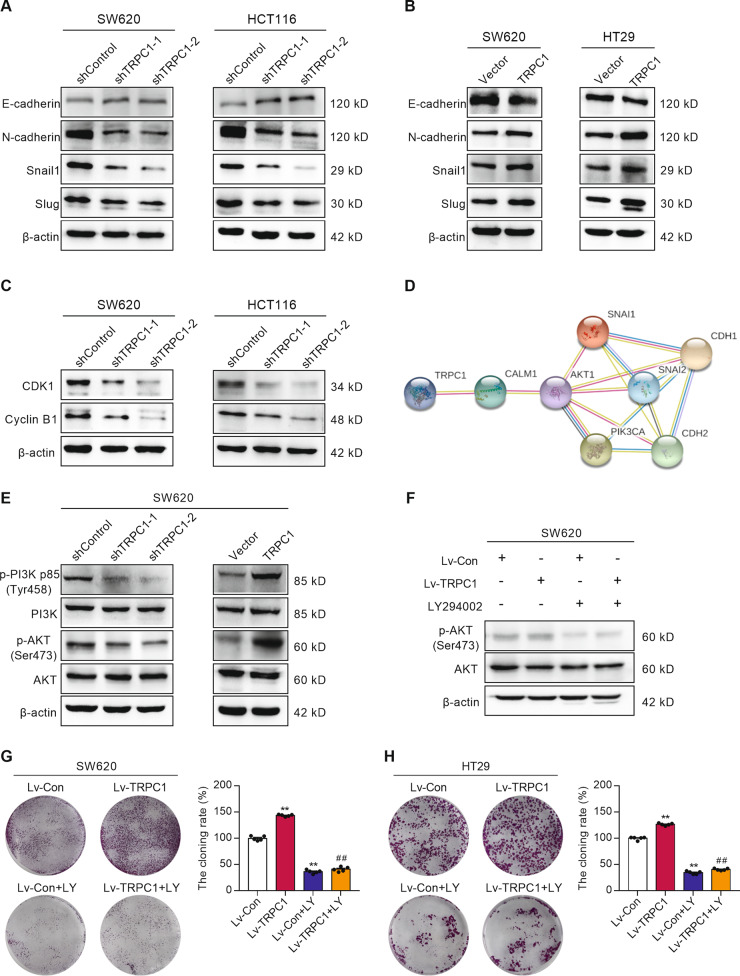
TRPC1 regulates the levels of multiple molecules involved in the cell cycle and EMT through the PI3K/AKT pathway in CRC cells. **A** The relative protein levels of EMT regulators, including E-cadherin, N-cadherin, Snail1, and Slug, detected in the SW620 and HCT116 cells with stably suppressed TRPC1 expression by Western blot analysis (*n* = 3). **B** The relative protein levels of EMT regulators, including E-cadherin, N-cadherin, Snail1, and Slug, detected in the SW620 and HT29 cells overexpressing TRPC1 by Western blot analysis (*n* = 3). **C** Knockdown of endogenous TRPC1 in SW620 and HCT116 cells reduced the protein levels of CyclinB1 and CDK1 (*n* = 3). **D** The visualization of protein–protein interaction of TRPC1 by using the STRING analysis. With the potential binding proteins of TRPC1, we combined the key proteins, related cycles, and EMT marker proteins in the development of colorectal cancer, and then screened them in the STRING database. KEGG pathway analysis showed that PI3K/AKT might be a significant downstream regulated signaling pathway of TRPC1 in cancer pathway (hsa05200:Pathways in cancer: *p* = 0.00031; hsa04151: PI3K/AKT signaling pathway: *p* = 0.011). **E** The levels of PI3K, phospho-PI3K (p-PI3K p85), AKT, and phospho-AKT were examined by Western blot analysis in the SW620 cells with silenced or enhanced TRPC1 (*n* = 3). **F** Effects of LY294002 on AKT activation in the SW620 cells with TRPC1 overexpression or vector by Western blot (*n* = 3). **G**, **H** LY294002 suppressed colony formation in the TRPC1 overexpressed CRC cells. Typical results are shown in the left panel along with the statistical analysis in the right panel (*n* = 5). **P < 0.01 vs. Lv-Con group; ^##^*P* < 0.01 vs. Lv-TRPC1 group.

To better understand the mechanism by which TRPC1 regulates the cell cycle progression and metastasis, we detected the expression of several key signaling molecules involved in these biological processes. Inhibition of TRPC1 elevated the protein levels of the epithelial marker E-cadherin/CDH1, but reduced the expression of mesenchymal markers, namely, N-cadherin/CDH2, Snail1, and Slug (Fig. 5A and Supplementary Fig. S5A). Contrarily, cells overexpressing TRPC1 showed a significant increase in the levels of N-cadherin, Snail1, and Slug, and a decrease of E-cadherin (Fig. 5B and Supplementary Fig. S5C). Meanwhile, the expressions of some vital cell-cycle regulators were detected by Western blot analysis. TRPC1 knockdown in the SW620 and HCT116 cells markedly reduced the expressions of CyclinB1 and CDK1, which promoted the G2 phase progression (Fig. 5C and Supplementary Fig. S5B). These data demonstrated that both cell cycle and EMT were TRPC1-regulated biological processes.

### TRPC1 boosts CRC progression through PI3K/AKT activation

To explore the molecular mechanisms underlying the oncogenic effect of TRPC1, the STRING protein–protein interaction analysis was used to find that TRPC1 was highly related to PI3K/AKT signaling pathway (Fig. 5D). Accumulating evidence supports a crucial role of PI3K/AKT signaling in regulating cell cycle progression, cell proliferation, and metastasis in a variety of tumors [20, 21]. Hence, the phosphorylated levels of PI3K and AKT were analyzed using Western blotting, confirming that TRPC1 knockdown significantly reduced their activation, whereas TRPC1 overexpression promoted their phosphorylation in the SW620 cells (Fig. 5E and Supplementary Fig. S5D). To identify the oncogenic effect of TRPC1 through activating PI3K/AKT signaling, HT29 and SW620 cells with accessorial expression of TRPC1 were further treated with PI3K inhibitor LY294002. After LY294002 treatment, the increased protein level of phospho-AKT induced by TRPC1 overexpression was significantly diminished (Fig. 5F and Supplementary Fig. S5E). Moreover, LY294002 reduced the promoted cell proliferation, invasion, and migration caused by TRPC1 overexpression to the normal inhibitor-treated levels in the SW620 and HT29 cells (Fig. 5G, H, and Supplementary Fig. S6A, B). And LY294002 could restore the expression level of EMT markers protein affected by TRPC1 overexpression (Supplementary Fig. S6C). In addition, when TRPC1 was highly expressed in SW620 cells, the autophagy marker LC3II/LC3I ratio tended to decrease, but the difference was not statistically significant (Supplementary Fig. S6D). The results showed that the activation of PI3K/AKT signaling pathway by TRPC1 had no significant effect on autophagy. Thus, our results indicate that PI3K was the crucial signaling molecule in TRPC1-regulated proliferation, invasion, and migration in CRC cells.

### TRPC1 enhances the interaction between CaM and PI3K in CRC cells

**Fig. 6:**
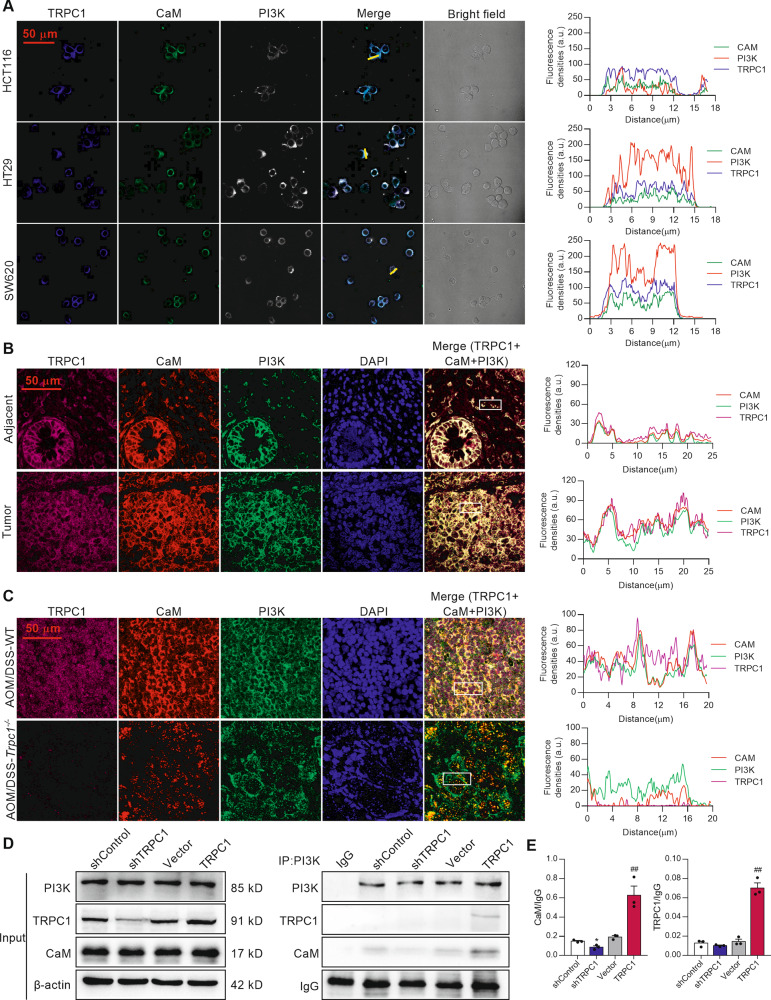
TRPC1 enhances the interaction between CaM and PI3K in colorectal cancer. **A** The colocalization of TRPC1, CaM, and PI3K in CRC cells using immunofluorescence analysis. The representative immunohistological staining of TRPC1, CaM, and PI3K in the HCT116, HT29, and SW620 cell lines (left panel) and the traces of fluorescence intensity spatial profiles (right panel). **B** The representative immunohistological staining of TRPC1, CaM, and PI3K in human colorectal tumor tissues and adjacent tissues from the local cohort (left panel) and the traces of fluorescence intensity spatial profiles (right panel). Nuclear were stained with DAPI. **C** *Trpc1* knockout inhibited the colocalization of CaM and PI3K. Left panel, the representative immunohistological staining of TRPC1, CaM, and PI3K in CRC tumor tissues in the AOM/DSS-WT and AOM/DSS-*Trpc1*^*-/-*^ mice. Right panel, the traces of fluorescence intensity spatial profiles. **D**, **E** The interaction of TRPC1, CaM, and PI3K p85 subunit was confirmed by co-immunoprecipitation assay in SW620 cells. Typical results are shown in the left panel along with the statistical analysis in the right panel (*n* = 3). *P < 0.05 vs. shControl group; ^##^*P* < 0.01 vs. vector group.

In normal colonic epithelial NCM460 cell, TRPC1 cannot directly interact with PI3K (Supplementary Fig. S6E). So, we further analyzed the CaM protein, which was shown as an important intermediate molecule between the TRPC1 and PI3K/AKT signaling pathway through the STRING analysis (Fig. 5D). Intriguingly, the immunofluorescence analysis demonstrated that TRPC1, CaM, and PI3K were highly colocalized in the cytoplasm of CRC cells (Fig. 6A), which was further verified in human CRC tumor tissues and adjacent tissues (Fig. 6B). Consistently, TRPC1 was highly expressed and distinctly colocalized with CaM and PI3K in the tumor tissues of AOM/DSS-induced WT mice, while the colocalization of CaM and PI3K was markedly receded in the tumor tissues of *Trpc1*^*-/-*^ mice (Fig. 6C).

A co-immunoprecipitation experiment was performed to further confirm the protein interaction of TRPC1, CaM, and PI3K. As predicted, there was obvious interaction of CaM and PI3K p85 subunit in the SW620 control cells. Importantly, this binding was distinctly disrupted by TRPC1 knockdown, whereas it was markedly increased in the TRPC1-transfected SW620 cells (Fig. 6D, E). Moreover, there was explicit binding between TRPC1 and PI3K p85 subunit in the SW620 cells with TRPC1 overexpression (Fig. 6D, E), indicating that TRPC1 promoted the interaction between CaM and PI3K by directly binding to CaM, and depletion of TRPC1 may attenuate such binding and then block the PI3K activation.

### The oncogenic effect of TRPC1 in CRC is attenuated by silencing CaM

**Fig. 7:**
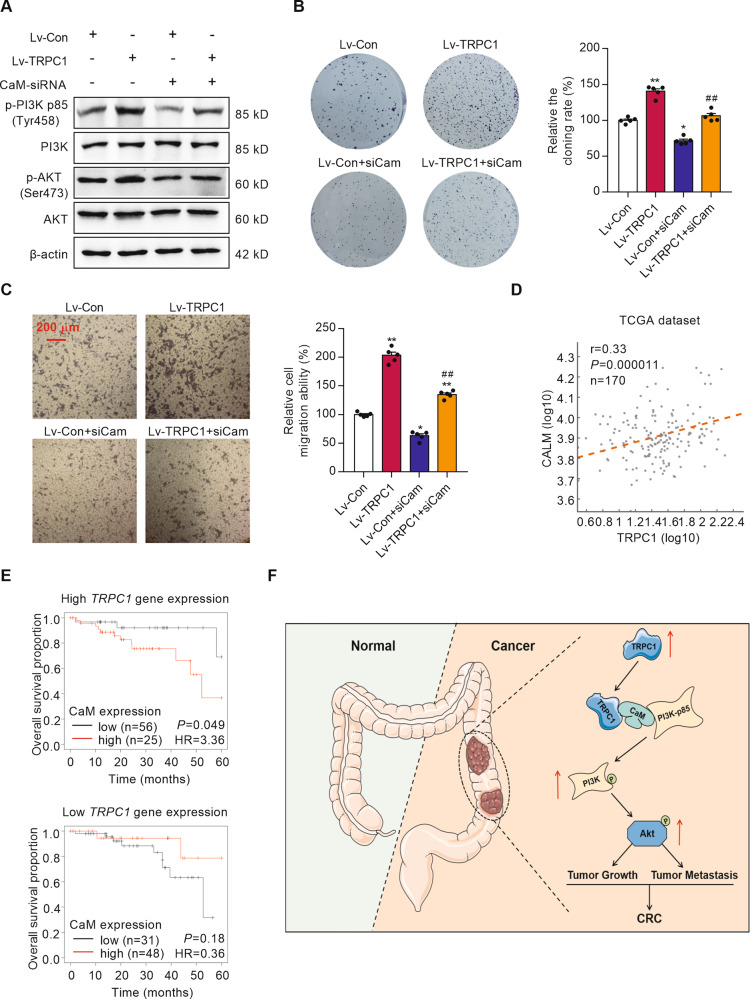
The effects of CaM in TRPC1-induced CRC progression. **A** Effects of CaM silencing on PI3K and AKT activation in the TRPC1 overexpressed or vector cells by Western blot analysis (*n* = 3). **B** Silence of CaM suppressed colony formation in the SW620 cells overexpressing TRPC1 (*n* = 5). **C** Silencing CaM restrained the migratory capabilities of the TRPC1 overexpressed SW620 cells as determined by the transwell migration assay (*n* = 5). *P < 0.05; **P < 0.01 vs. Lv-Con group. ##*P* < 0.01 vs. Lv-TRPC1 group. **D** The *TRPC1* mRNA expression was positively correlated with *CALM1* mRNA expression (*n* = 170; *P* < 0.0001, Pearson correlation coefficient analysis). **E** Kaplan–Meier curves of overall survival of CRC patients from the integrated cohort and stratified by *TRPC1* expression (*N*_high_ = 81, *N*_low_ = 79). **F** Schematic representation of the proposed mechanisms of TRPC1 functions during tumor growth and metastasis cascade in CRC. The enhanced expression of TRPC1 in CRC enhances the interaction between CaM and the p85 subunit of PI3K by directly binding to CaM, which in turn accelerates the phosphorylation of PI3K and AKT, thereby activating the PI3K/AKT signaling cascade and then promoting tumor growth and metastasis in CRC.

To test whether TRPC1 regulates PI3K/AKT signaling through direct interaction with CaM, we silenced CaM in the TRPC1-transfected SW620 cells. The successful silencing was identified by a Western blot analysis (Supplementary Fig. S2C). CaM silencing significantly diminished the increased protein levels of the phospho-PI3K and phospho-AKT induced by TRPC1 overexpression, but not the total PI3K and AKT protein levels (Fig. 7A and Supplementary Fig. S7A). To further validate this function, cellular proliferation and migration were measured in the siCaM-treated CRC cells. The relative colony formation rate and migration ability of siCaM-treated TRPC1-overexpressed SW620 cells were markedly decreased to 75.8% and 65.8% of the TRPC1-transfected cells, respectively, demonstrating that CaM knockdown had attenuated the oncogenic effect of TRPC1 in CRC cells (Fig. 7B, C). And CaM silencing did not significantly diminish the reduced protein levels of the phospho-PI3K and phospho-AKT induced by TRPC1-knockdown in SW620 cell (Supplementary Fig. S7B). The relative colony formation rate and migration ability of siCaM-treated TRPC1-knockdown SW620 cells were not markedly inhibited compared with the TRPC1-knockdown cells (Supplementary Fig. S7C, D). Additionally, the important scaffold role of CaM in TRPC1-regulated CRC progression was analyzed in the TCGA dataset. There was a positive association between *TRPC1* mRNA and *CALM1* mRNA expressions (Fig. 7D). Furthermore, we analyzed the overall survival for CaM expression in patients stratified according to high (*n* = 81) or low (*n* = 79) *TRPC1* expression. It is worth mentioning that CaM expression had no prognostic value in patients with low *TRPC1* expression, whereas high CaM expression was associated with lower overall survival in patients with high levels of *TRPC1* (Fig. 7E). Cbioportal database analyzed the overall survival for CaM expression in patients stratified according to TRPC1 expression in both PI3K mutation and non-mutation, which were not significantly different (Supplementary Fig. S7E). Collectively, our data provided evidence that TRPC1 exhibits its oncogenic effect by activating CaM-mediated PI3K/AKT signaling axis.

## Discussion

TRPCs, besides controlling cation entry, regulate important cellular functions, including cell proliferation, survival, and migration [13, 22]. Recently, the role TRPCs play in tumors is gradually being discovered [14]. However, there has been little research on the effects of TRPCs regulation on CRC progression and the anti-tumor mechanism of its action remains limited. In this study, we revealed the role and mechanism of TRPCs in CRC progression. Among the six human TRPC subunits, TRPC1 is significantly upregulated in CRC tissues and high levels of TRPC1 significantly correlate with the aggressive characteristics of CRC as well as poor survival in CRC patients. KRAS, BRAF, and PI3K gene mutations promote the occurrence and development of CRC by activating downstream signaling pathways. We also analyzed TRPC1 expression in function of KRAS, BRAF, and PI3K mutational status with the Cbioportal database (data from TCGA). The results show that TRPC1 expression was decreased in CRC patients with KRAS mutation, but no significant difference was observed in PI3K and BRAF mutation conditions (Supplementary Fig. S1A). Therefore, we modulated TRPC1 level in different CRC cell lines (HCT116 cell line with KRAS mutation, SW620 cell line with KRAS and PIK3CA mutations, and HT29 cell line with BRAF and PIK3CA mutations) and found that the knockdown of TRPC1 reduced cell proliferation, cell-cycle progression, invasion, and migration in vitro, and inhibited tumor growth of the xenograft mice models. Importantly, depletion of *Trpc1* significantly suppressed tumorigenesis in vivo. Overexpression of TRPC1 promoted the proliferation, invasion, and migration of colorectal cancer cells. In addition, both in KRAS, BRAF as well as PI3K mutant and non-mutant CRC cell lines, the change of TRPC1 expression had significant effects on the proliferation, invasion, and migration. Hence, TRPC1 has the independent pleiotropic promoting effects on the genesis, tumor growth, and metastasis of CRC.

Recently, growing evidence suggests that TRPC1 acts as pro-oncogenes in some kinds of malignant tumors. For instance, silencing TRPC1 is able to inhibit the proliferation of hepatocellular carcinoma cells [23], decrease the invasion, migration, and proliferation of thyroid cancer cells via reducing HIF-1α expression [24]. A study for CRC also showed that transcript levels of *TRPC1* are upregulated in the HT29 cell line compared with normal cell NCM460 [25]. In the present study, we demonstrated that TRPC1 expression in human CRC tissues was much higher than that in adjacent tissues and negatively correlated with prognosis of CRC patients. Moreover, high levels of TRPC1 positively correlated with TNM stage and tumor metastases in CRC patients. Thus, the meaning of TRPC1 in CRC pathophysiology is strongly suggested. Noticeably, despite TRPC1 being structurally homologous to other subunits of the TRPC family, their distribution and function in various systems are not the same [26]. That may explain why the mRNA expression of other *TRPCs* are not significantly different between colorectal tumor tissues and adjacent normal tissues.

To further explore the oncogenic role of TRPC1 in CRC, we performed gain of function and loss of function experiments in CRC cell lines and found that TRPC1 knockdown dramatically blocked cell proliferation, migration, and invasion in vitro as well as tumorigenicity in vivo. It has been defined that unscheduled proliferation is often induced by cell cycle defects as well as dysregulation of cyclins and cyclin-dependent kinases (CDKs) complexes [27]. CyclinB1 and CDK1 are important components of the maturation-promoting factors. CDK1 activity requires the binding of a regulatory subunit called Cyclin B1 and the activation of the Cyclin B1/CDK1 complex is responsible for the transition from the G2 to M phase [28]. In this study, TRPC1 knockdown markedly reduced the expressions of CDK1 and CyclinB1, and further caused the arrest of the G2/M transition, which might decline cell proliferation. The development of tumors can be conceptually simplified into two major phases: the first is the genesis, and the second is the growth. Our study firstly discovered that *Trpc1* knockout significantly reduced the tumor incidence rate in CRC mice model, suggesting that TRPC1 plays an incitant role in the genesis of CRC. Furthermore, tumor metastases are the cause of 90% of cancer-related deaths [29]. Our findings provided evidence that TRPC1 promoted cell migration and invasion in CRC cells and lung metastasis in mice. TRPC1 exerted its pro-metastatic effect by promoting EMT, as indicated by the loss of the epithelial marker, E-cadherin, and increased the expression of the mesenchymal markers, N-cadherin, Snail1, and Slug, thereby favoring a mesenchymal phenotype that enables increased cell migration and invasion [30, 31]. Collectively, TRPC1 functions as a versatile oncogene in CRC by promoting tumor genesis, growth, and metastasis.

Recent studies have suggested that changes in the intracellular Ca^2+^ homeostasis may contribute to critical cancer hallmarks, such as enhanced cell proliferation, migration, invasion, and survival [32]. TRPC1 protein is referred to mediate store-operated Ca^2+^ entry (SOCE), a major mechanism controlling basal Ca^2+^ levels and intracellular Ca^2+^ store refilling, which is dramatically enhanced while Ca^2+^ stores are partially empty in CRC cells [33]. In a previous study, Sajida Ibrahim et al. found that colon cancer cells display an abnormal expression of SOCE molecular players including ORAI1, ORAI3, STIM1, and TRPC1 [34]. Although SOCE is largely upregulated in the HT29, SW480-ADH, and SW480-R CRC cell lines, TRPC1 silencing did not affect SOCE level in the HT29 cell [25]. In our study, calcium imaging assay showed that TRPC1-knockdown could only inhibit the calcium influx in SW620 cells, but had no significant effect on the calcium influx in HCT116 and HT29 cells (Supplementary Fig. S8AB and C). But, our experiment showed that TRPC1-knockdown significantly affected the proliferation, invasion, and migration of SW620 and HCT116 cells. Additionally, immunofluorescence assay showed that, besides cytomembrane, TRPC1 also located in the cytoplasm of colorectal cancer cell lines HT29, SW620, and HCT116, which highlighted the possible role of TRPC1 as the cytoplasmic protein in CRC cells (Supplementary Fig. S8D). These results indicate that TRPC1 does not play a major role in the SOCE of CRC cells, and that TRPC1 may perform other functions in CRC progression in addition to acting as a channel protein.

To extensively study the mechanisms responsible for TRPC1-mediated CRC progression, we performed STRING analysis and found that TRPC1 is highly correlated with the PI3K/AKT signaling cascade. It is well-known that the PI3K/AKT pathway plays a crucial role in cell growth and metabolism ultimately influencing the proliferation, invasion, and metastasis of several types of cancer cells [35]. Inhibition of the PI3K/AKT signal could decrease the levels of CyclinB1 and CDK1, thereby causing the G2/M cell cycle arrest in HCC cells and bladder cancer cells [36, 37]. Moreover, a bulk of evidence supports the crucial role that the PI3K/AKT signal plays on EMT by downregulating E-cadherin as well as upregulating N-cadherin, Snail, and Slug [38, 39]. Our findings showed that the PI3K/AKT pathway was activated since p-PI3K and p-AKT were upregulated by TRPC1. We further verified that the upregulation of CDK1, CyclinB1, N-cadherin, Snail, and Slug as well as the downregulation of E-cadherin by TRPC1, have resulted in the promotion of cell proliferation, invasion, and migration through the PI3K/AKT pathway by using PI3K inhibition. Thus, TRPC1 exerts oncogenic effects in CRC through activating PI3K/AKT signaling axis.

Calmodulin mainly functions as a modulator for the activity of protein kinases and phosphatases, as well as other signaling proteins [17]. Certain evidence suggests that TRPC1 participates in a variety of pathological and physiological processes by regulating CaM [40]. In our study, by using siRNA of CaM, the increased protein levels of p-PI3K and p-AKT induced by TRPC1 overexpression was significantly decreased. Moreover, siCaM could reduce the promoted cell proliferation and migration caused by TRPC1 overexpression close to the normal levels in the SW620 cells. A molecular structure docking assay indicated that activated CaM can specifically bind to the sh2 domain of PI3K regulatory subunit p85, further removing the inhibition of p85 subunit on the catalytic subunit p110, which in turn activates PI3K signaling [41]. Moreover, the combination of CaM and p85 subunit of PI3K were discovered by a co-IP experiment in bovine aortic endothelial cells [42]. The present study is the first of its kind to reveal that TRPC1, CaM, and PI3K were co-located in CRC cells and tumor tissues using immunofluorescence. Further co-IP assay confirmed that TRPC1 overexpression in CRC cells enhanced the interaction between CaM and PI3K p85 subunit. Noticeably, the interaction between TRPC1 and PI3K p85 subunit were not shown in CRC cells, but appeared in the TRPC1-transfected CRC cells, indicating that CaM is a connexin between TRPC1 and PI3K. Maxime Guéguinou et al. reported that the TRPC1-knockdown inhibited SOCE levels, which reduces p-AKT protein levels and effects the CRC cell migration. While AKT is not a Ca^2+^ binding protein, the specific data and evidence supporting how does the Ca^2+^ mediated by this channel complex activates AKT pathway has not been provided and explained [43]. Our findings suggest that TRPC1 can promote the proliferation, invasion, and migration of CRC cells by Ca^2+^ independent pathway, which directly binds to CaM to promote the binding between CaM and PI3K, and then activate the PI3K/AKT signaling pathway.

CaM, composed of N- and C-terminal domains connected with a flexible linker, has two EF-hand motifs in each domain [18]. It has been reported that a direct interaction between TRPC1 and CaM exists, and the binding domain between CaM and TRPC1 was the EF-hand domain [44, 45], which is different from the binding domain (residues 118–140) between CaM and PI3K [42]. Our study also indicated the three molecular complex between TRPC1, CaM, and PI3K in CRC cells. Based on the different binding sites of the interacting proteins, human TRPC1 (homologous modeling according to TRPC4 protein), CaM, and PI3K proteins were analyzed using the AutoDock 4.2 software to demonstrate the probable binding mechanism by which TRPC1 regulates PI3K (Supplementary Fig. S9). Although the structures of the TRPC family are relatively conservative, the current docking analysis of the interaction site based on homologous modeling TRPC4 protein is limited, since the crystal structure of TRPC1 protein has not been resolved. A direct molecular function of TRPC1 in enhancing the interaction between CaM and PI3K still needs further investigation.

In summary, our findings provided evidence that TRPC1 upregulation is a common event in colorectal cancer and an altered expression of TRPC1 is important for the genesis, tumor growth, and metastasis of CRC. Moreover, our data demonstrate that TRPC1 enhances the interaction between CaM and the PI3K p85 subunit by directly binding to CaM, which further activated the PI3K/AKT and its downstream signaling molecules implicated in cell cycle progression and epithelial-mesenchymal transition (Fig. 7F). Thus, targeting TRPC1 might be a novel strategy for hindering uncontrolled growth and metastasis in PI3K/AKT-addicted colorectal cancer.

## Materials and methods

Additional information on reagents and experimental procedures are described in the Supplementary Materials and Methods.

### Statistical analysis

Experiments were performed in at least three or five independent replicates. Data are represented as mean ± S.E.M. To analyze all data for statistical significance, SPSS 22.0 (IBM Corp., USA) and GraphPad Prism 8.0 (GraphPad Software, Inc., CA) software were used. The independent or paired two-tailed Student *t-*test was used to compare the difference between the two groups, and one-way ANOVA was used for multi-group comparisons. Survival curves were evaluated by Kaplan–Meier method and log rank test. The Pearson correlation coefficient was used to evaluate the correlation between the *TRPC1* and *CALM1* expressions in the clinical samples. The difference in cell viability and tumor growth rate was determined by repeated-measures ANOVA. The value of *P* < 0.05 was considered statistically significant.

## Supplementary information

Supplementary Fig S1

Supplementary Fig S2

Supplementary Fig S3

Supplementary Fig S4

Supplementary Fig S5

Supplementary Fig S6

Supplementary Fig S7

Supplementary Fig S8

Supplementary Fig S9

Supplementary Materials and Methods

## Data Availability

The datasets generated and/or analyzed during the current study are available from the corresponding author on reasonable request.
