# Supplementary figures and images for: TRPC1 promotes the genesis and progression of colorectal cancer via activating CaM-mediated PI3K/AKT signaling axis
Source: Oncogenesis. 2021 Oct 12;10(10):67. doi: 10.1038/s41389-021-00356-5 (PMC8511127; doi:10.1038/s41389-021-00356-5)

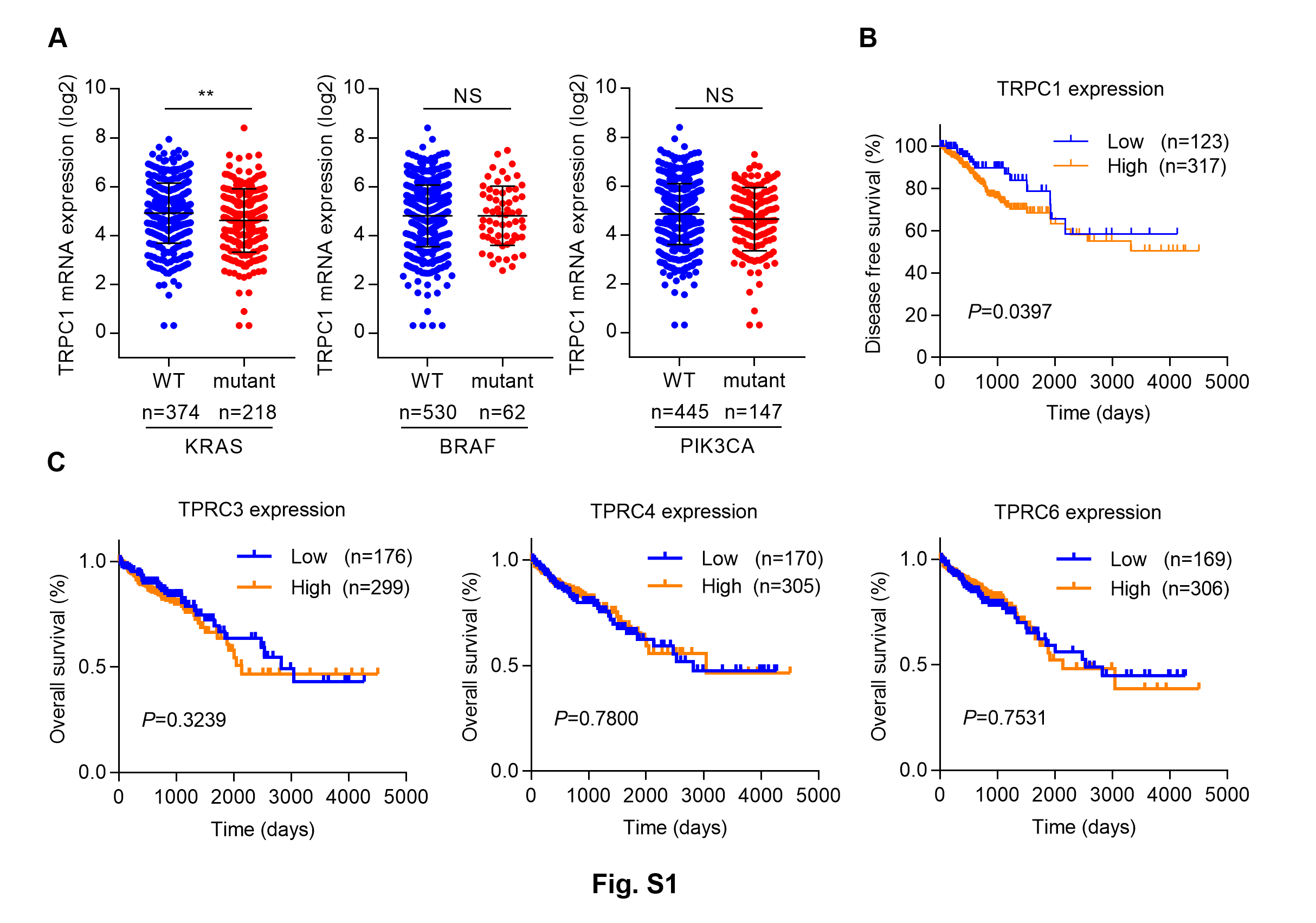

Supplement: Supplementary file 1 — Supplementary Fig S1 [file 41389_2021_356_MOESM1_ESM.tif]

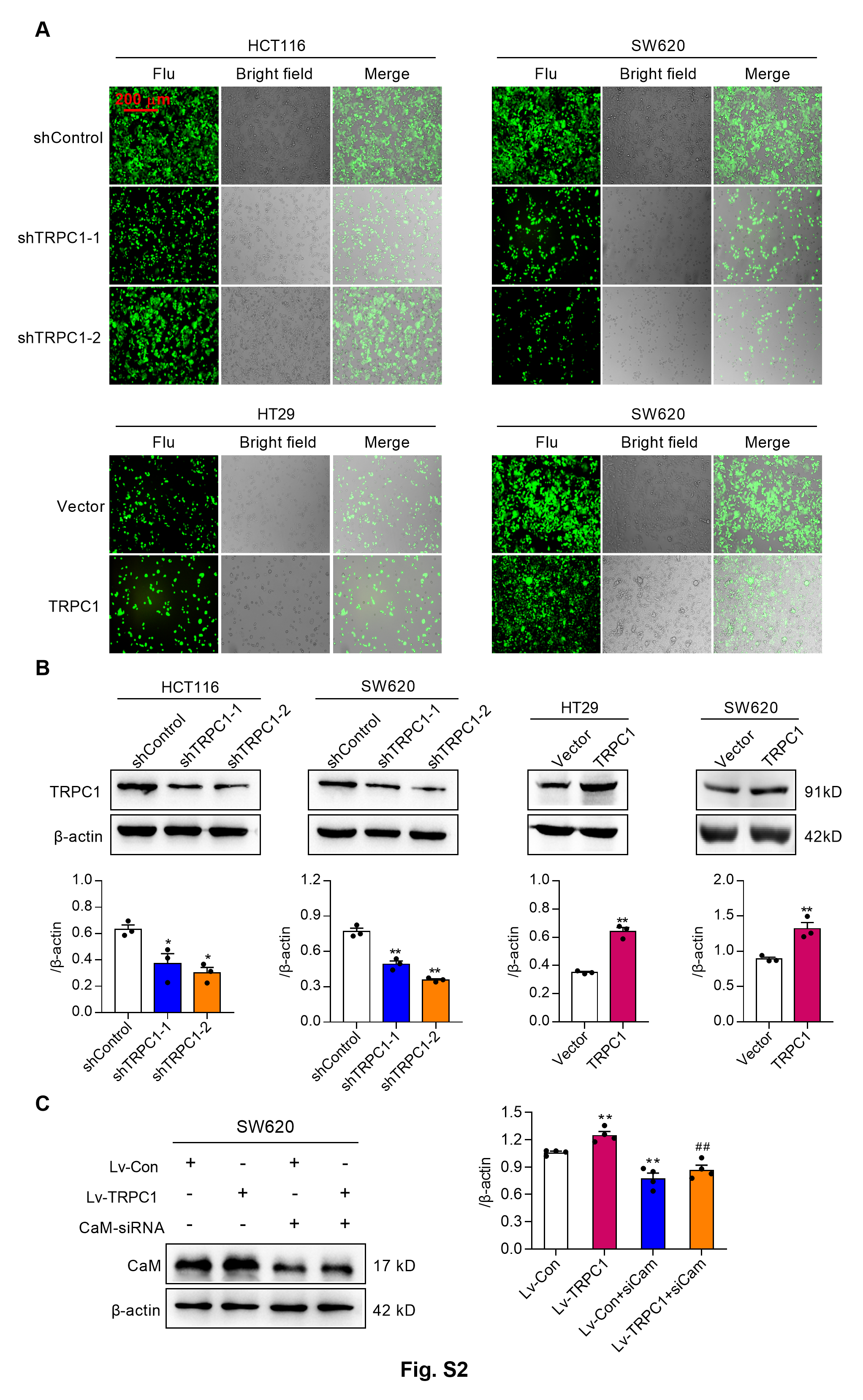

Supplement: Supplementary file 2 — Supplementary Fig S2 [file 41389_2021_356_MOESM2_ESM.tif]

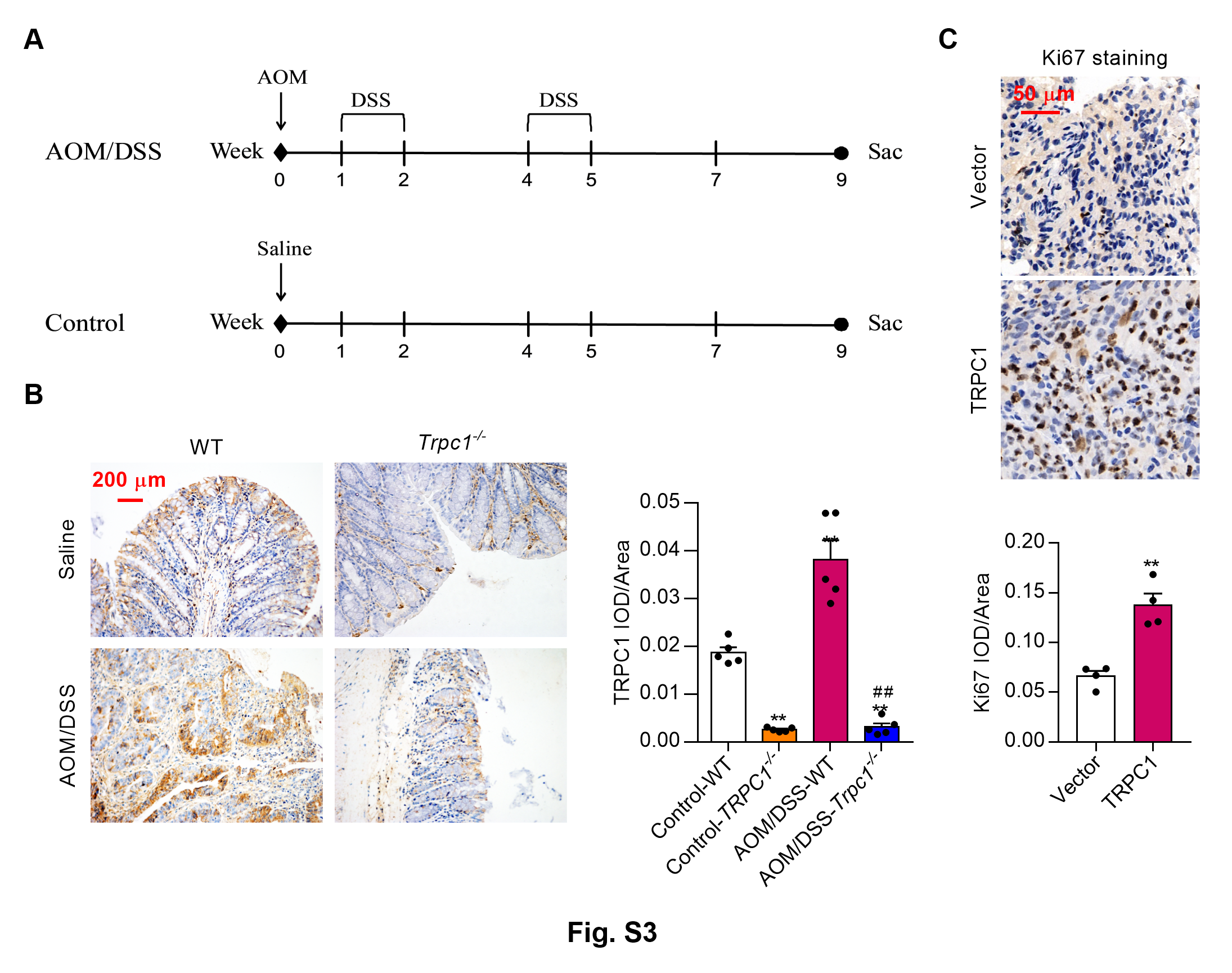

Supplement: Supplementary file 3 — Supplementary Fig S3 [file 41389_2021_356_MOESM3_ESM.tif]

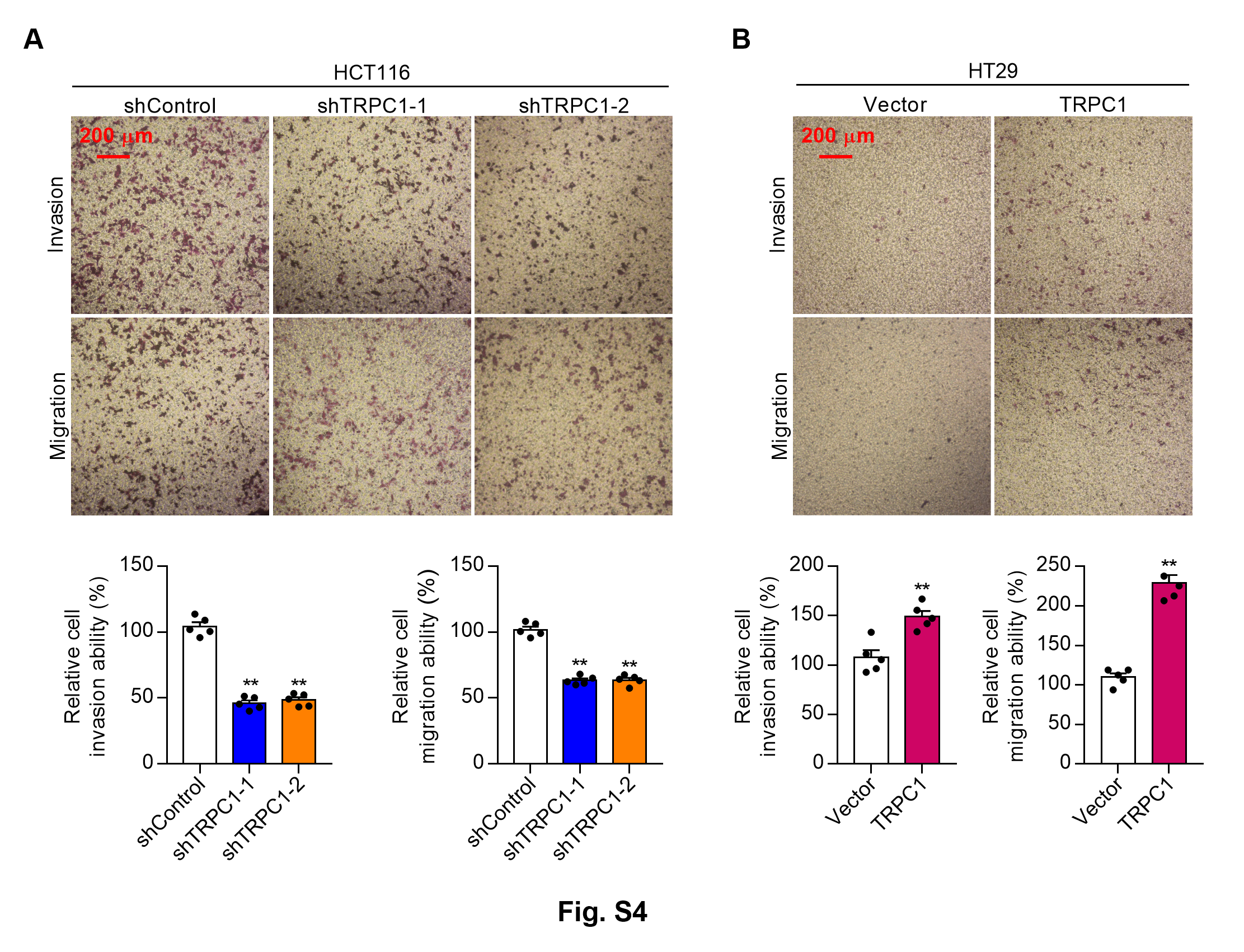

Supplement: Supplementary file 4 — Supplementary Fig S4 [file 41389_2021_356_MOESM4_ESM.tif]

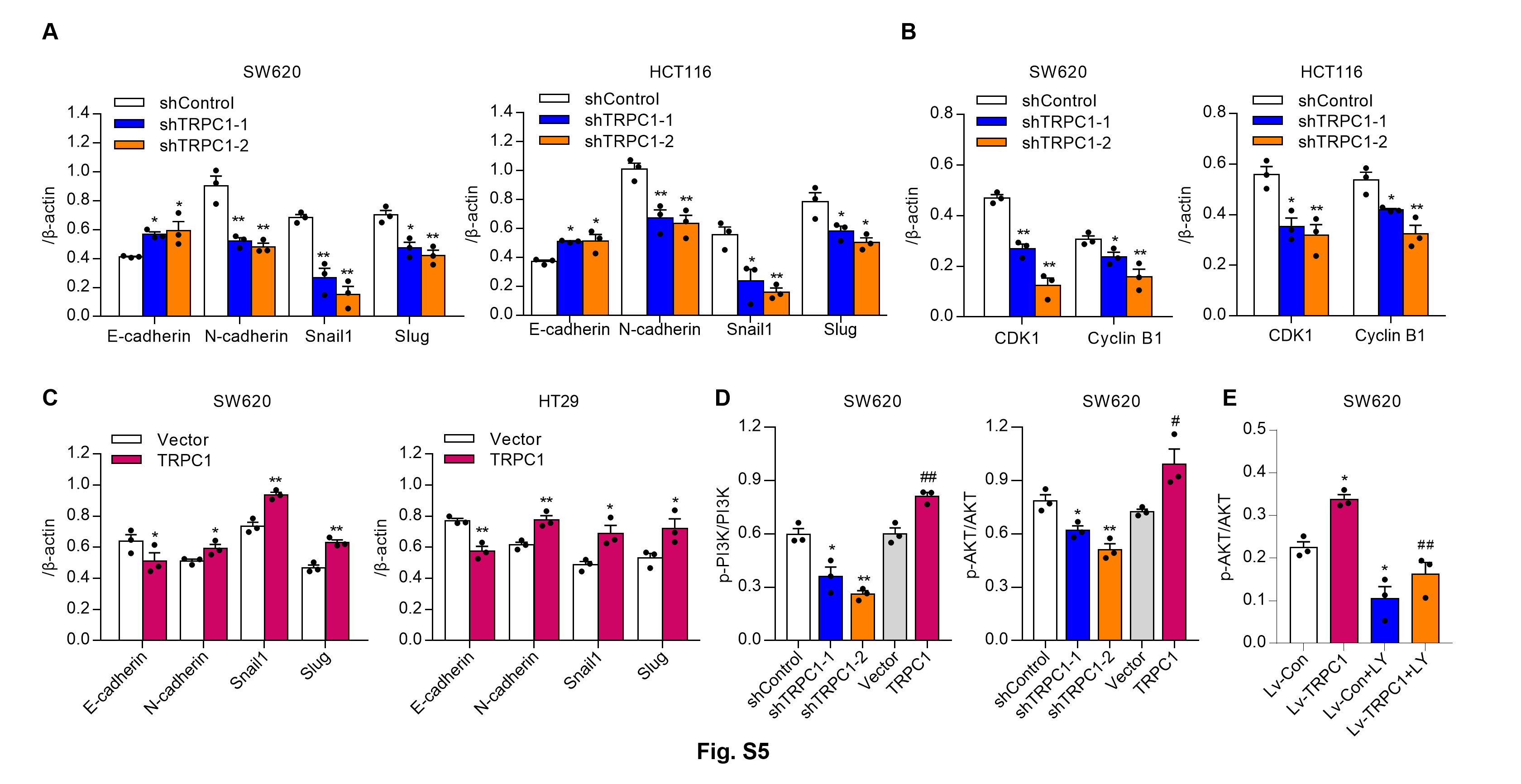

Supplement: Supplementary file 5 — Supplementary Fig S5 [file 41389_2021_356_MOESM5_ESM.tif]

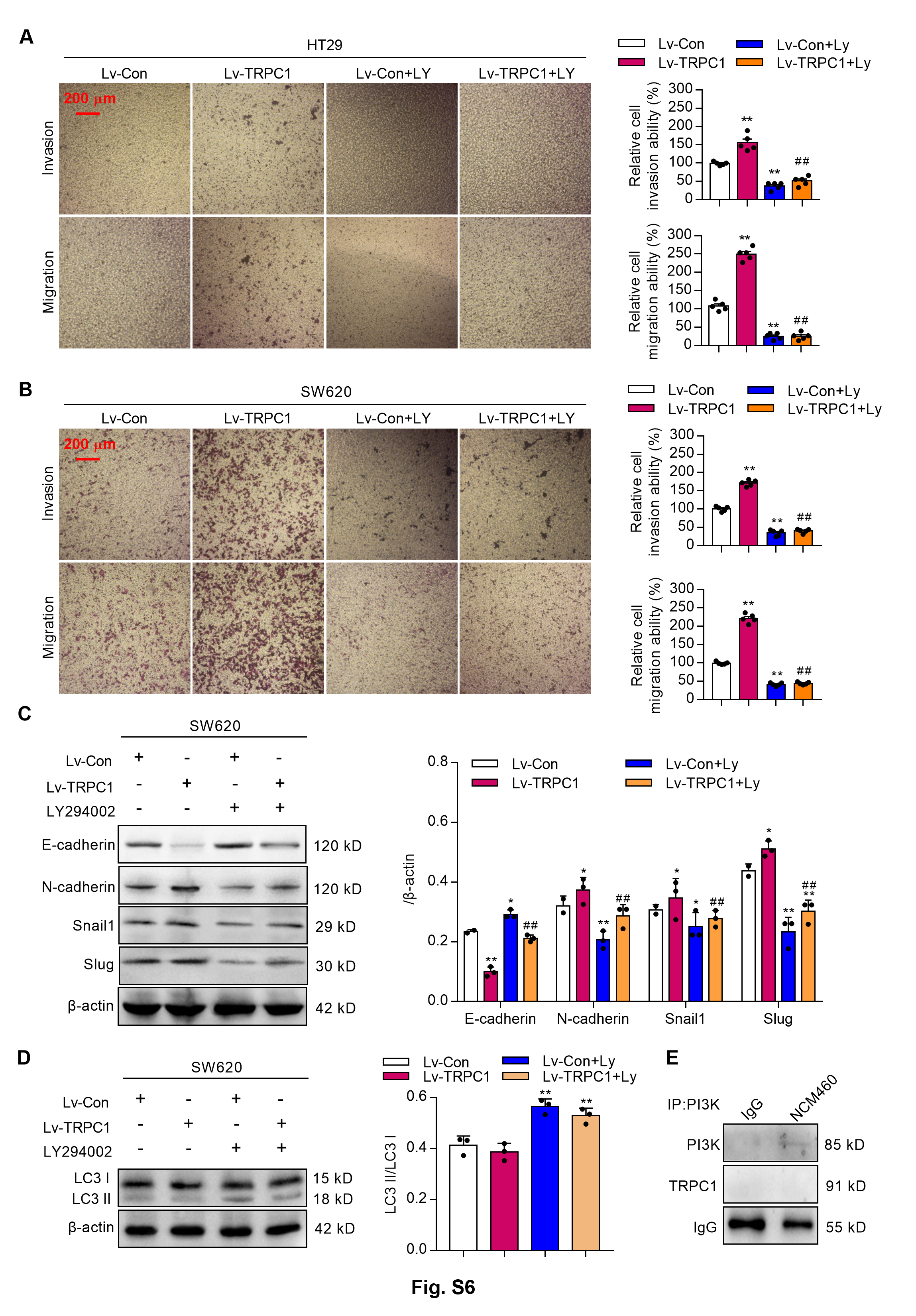

Supplement: Supplementary file 6 — Supplementary Fig S6 [file 41389_2021_356_MOESM6_ESM.tif]

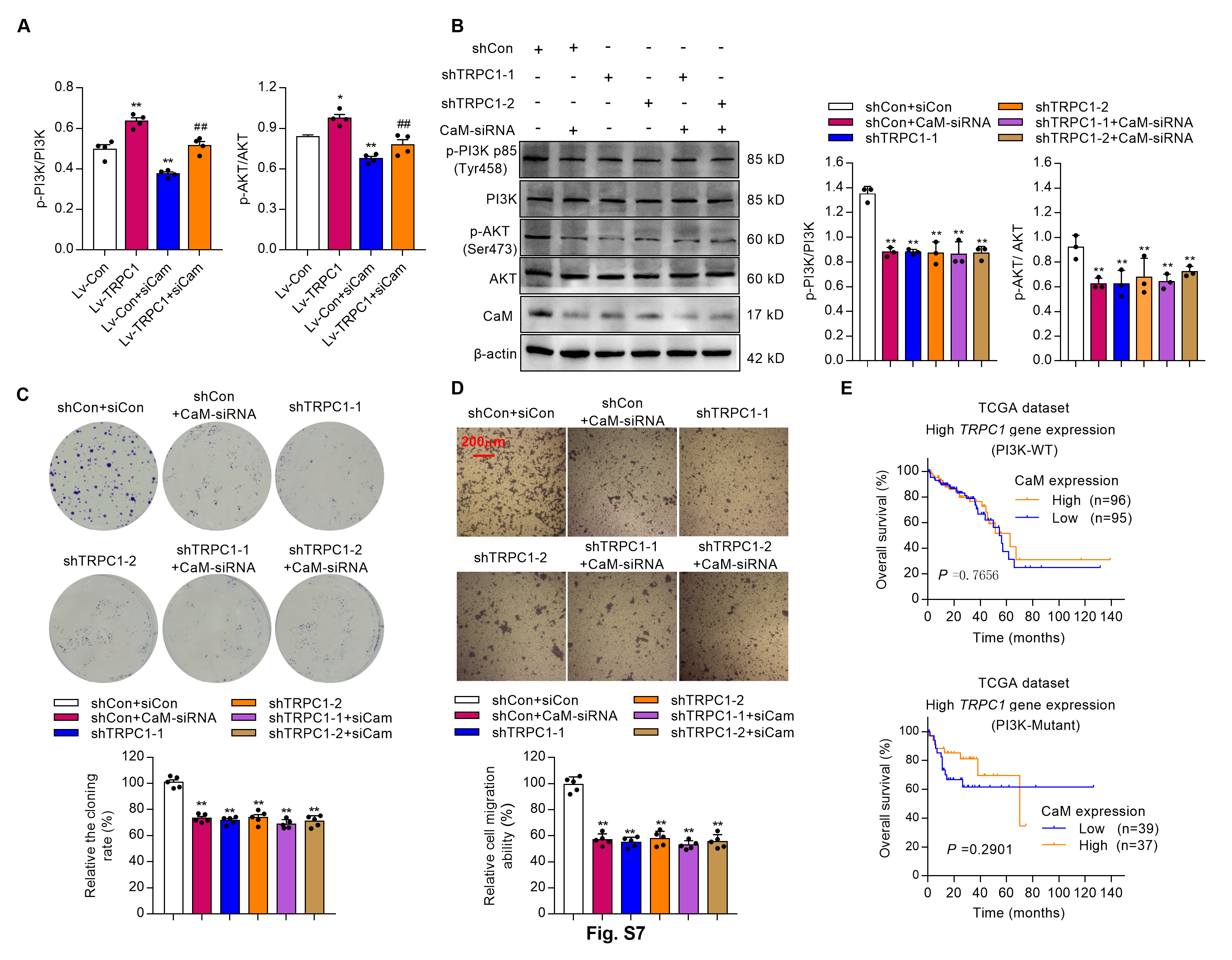

Supplement: Supplementary file 7 — Supplementary Fig S7 [file 41389_2021_356_MOESM7_ESM.tif]

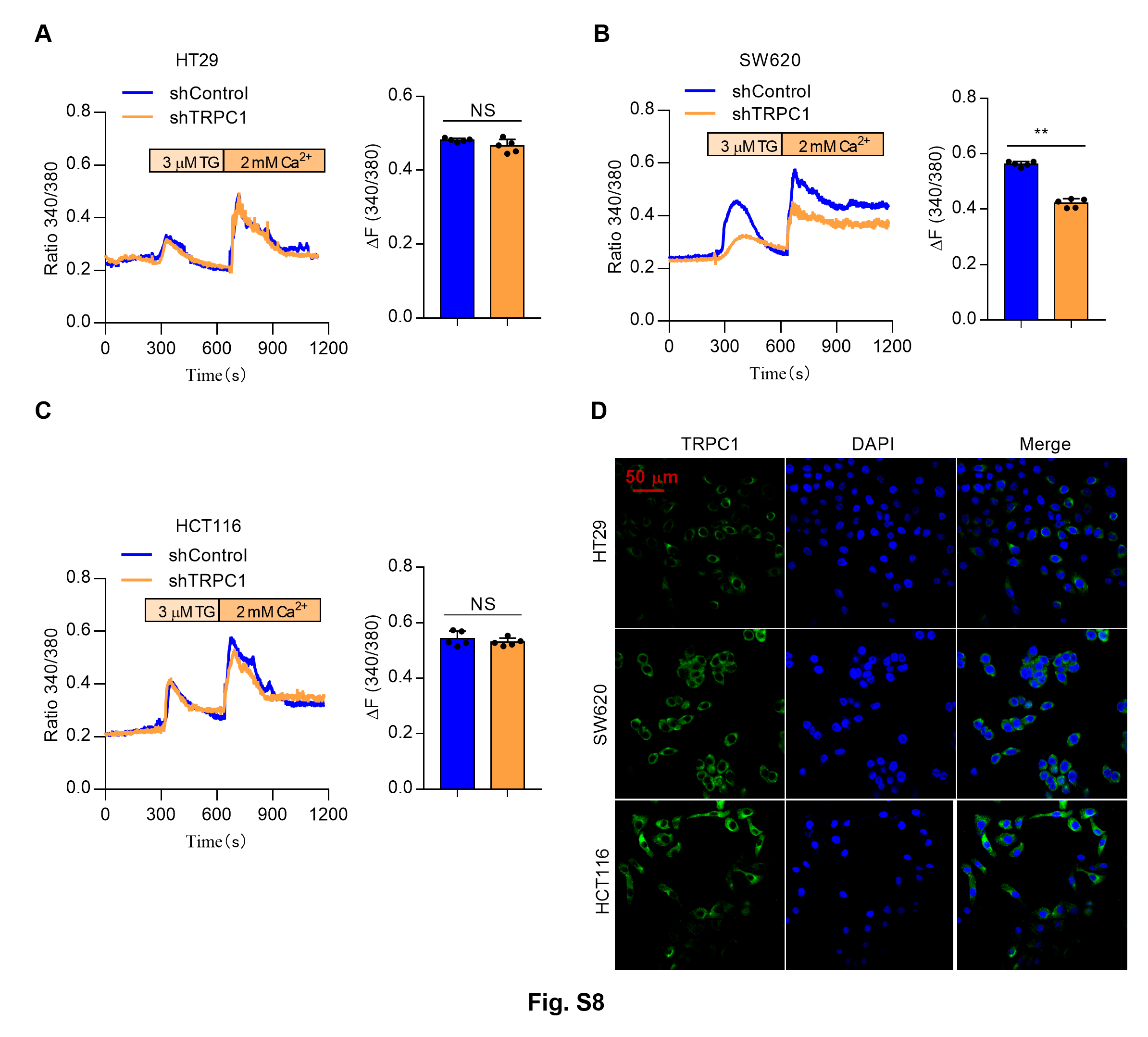

Supplement: Supplementary file 8 — Supplementary Fig S8 [file 41389_2021_356_MOESM8_ESM.tif]

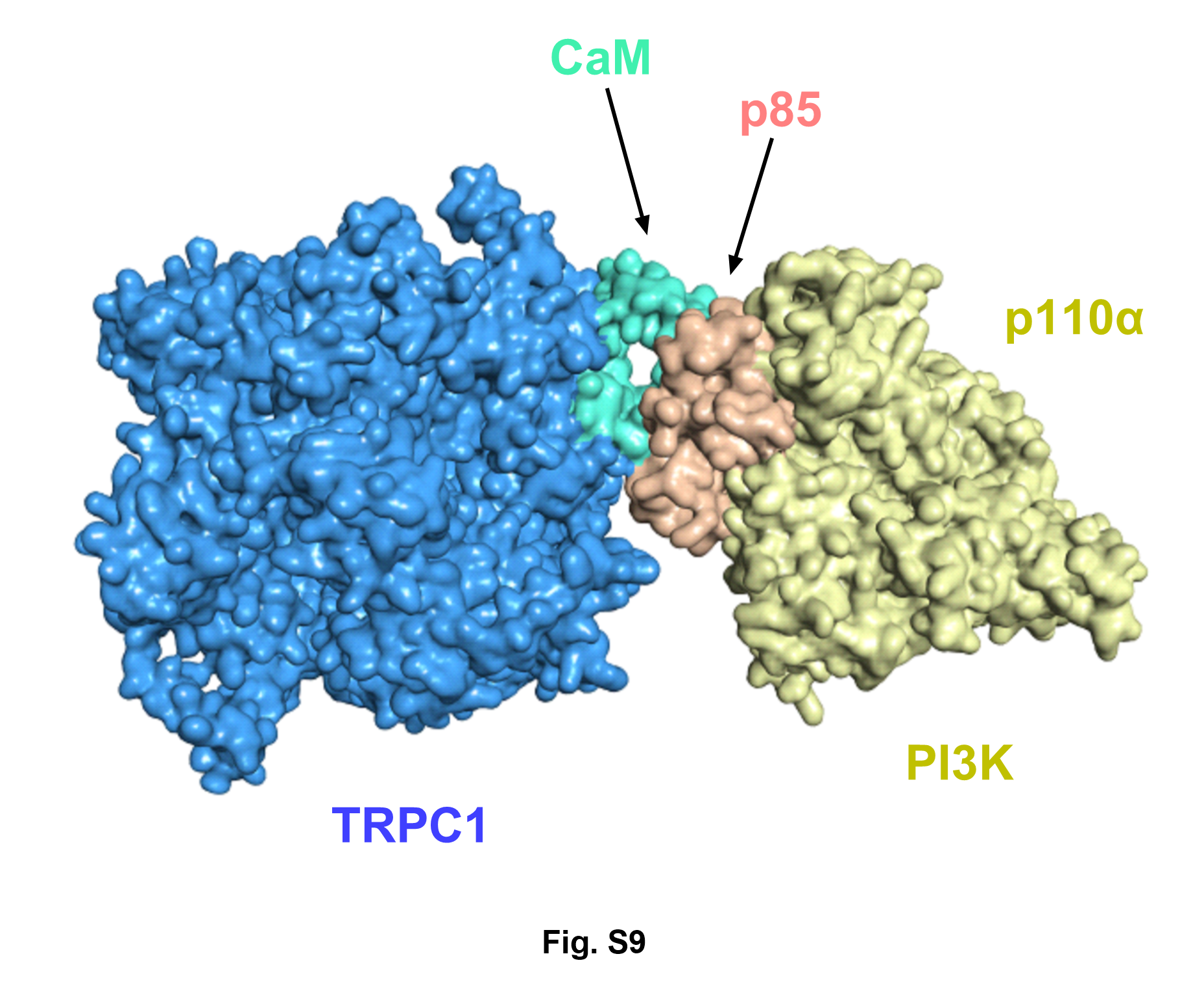

Supplement: Supplementary file 9 — Supplementary Fig S9 [file 41389_2021_356_MOESM9_ESM.tif]
