# Supplementary material for: TRPC1 promotes the genesis and progression of colorectal cancer via activating CaM-mediated PI3K/AKT signaling axis
Source: Oncogenesis. 2021 Oct 12;10(10):67. doi: 10.1038/s41389-021-00356-5 (PMC8511127; doi:10.1038/s41389-021-00356-5)
Supplement: Supplementary file 10 — Supplementary Materials and Methods [file 41389_2021_356_MOESM10_ESM.doc]

**Supplementary Materials and Methods**

**Public database**

Genetic data for normal and tumor tissues were downloaded from the Gene Expression of Normal and Tumor tissues 2 (GENT2) database (http://gent2.appex.kr/gent2/), including 17 gene chips which covered 287 normal colorectal tissues and 1994 CRC tissues. The Cancer Genome Atlas (TCGA) mRNA expression data of CRC were accessed via the National Cancer Institute GDC Data Portal website (https://portal.gdc.cancer.gov/) and 459 cases（Inclusion criteria were patients with clinical information） were included for analysis. Survival analysis of CRC patients from TCGA database was performed on Oncolnc website (https://www.oncolnc.org/). The STRING database (https://string-db.org/) was used to look for protein-protein interactions of TRPC1. The overall survival for CaM expression in patients stratified according to TRPC1 expression were analyzed using Kmplot database (https://www.kmplot.com/). Cbioportal database (https://www.cbioportal.org/datasets).

**Human tissue samples**

Human CRC tissue microarray (CRC-001A.B) was obtained from Xijing Hospital, Xi'an, China. This microarray included 104 colorectal tumor tissues and 13 adjacent normal tissues. Samples were confirmed by immunohistochemical methods and staged according to the tumor-node-metastasis classification system. This study was carried out in accordance with the Declaration of Helsinki and approved by the Ethics Committee of First Affiliated Hospital of the Fourth Military Medical University (FMMU, No. KY20194099).

**Cell lines and cell culture**

Colorectal cancer cell lines HCT116, HT29, SW620 and CaCo2 (ATCC) were cultured in RPMI1640 (HyClone, USA) mingled with 10% fetal bovine serum (FBS) (Gibco, USA) and 1% penicillin/streptomycin. The human colonic epithelial cell NCM460 (ATCC) was maintained in DMEM (HyClone, USA) supplemented with 10% FBS and 1% penicillin/streptomycin. All cells were incubated at 37°C in a 5% CO_2_ atmosphere. All cells were validated to be free of *Mycoplasma* by using Mycoprobe Mycoplasma Detection Kit (R&D System, USA).

**Animals**

C57BL/6J wild-type (WT) mice were obtained from the Laboratory Animal Center of the FMMU. Athymic Balb/c nude mice were purchased from the Charles River Laboratory (Beijing, China). *Trpc1* knockout mice were purchased from the Jackson Laboratory (37347-JAX). Mice were raised in a specific pathogen-free conditions. Experiments were performed in accordance with China animal welfare guidelines and were approved by the relevant authorities (Approval Form of IEC, First Affiliated Hospital of Fourth Military Medical University).

**Construction of stable cell lines**

To obtain stable cell lines overexpressing TRPC1, the HT29 and SW620 cells were infected with the lv-TRPC1 or lv-Control viruses (GeneCopoeia, Inc., USA). To observe the knockdown effects of TRPC1, the SW620 and HCT116 cells were transfected with the shRNA-TRPC1 or control virus purchased from GeneCopoeia. All cells were transfected according to the manufacturer's protocol. The Catalog No. of TRPC1 lentivirus (lv-TRPC1, lv-Control, shRNA-TRPC1, and shControl) are as follows, shTRPC1 #1: HSH061231-LVRU6GP-b; shTRPC1 #2: HSH061231-LVRU6GP-c; shControl: CSHCTR001-LVRU6GP; lv-TRPC1: EX-Z0808-Lv201; lv-Control: EX-NEG-Lv201.

**RNA interference**

Short interfering RNA (siRNA) oligonucleotide duplexes targeting CaM used in this study were synthesized and purified by HanBio (Shanghai, China). The sequences of siCaM are as follows: Forward primer 5’- GCUGAUGGUAAUGGCACCATT -3’; Reverse primer 5’- UGGUGCCAUUACCAUCAGCTT -3’. The siRNA transfections of cancer cells were performed using Lipofectamine 3000 (Thermo Fisher Scientific, USA) according to the manufacturer’s instructions, and the knockdown efficiency was verified by Western blot analysis.

**Azoxymethane (AOM) / Dextran sodium sulfate (DSS) - induced CRC mouse model**

Male C57BL/6J WT mice and *Trpc1*^-/-^ mice weighing 18 to 22 g were randomly divided into a control group and a model group (5/group, Male). To induce colorectal cancer, mice in the model group were intraperitoneally injected with AOM (10 mg/kg, Sigma-Aldrich, USA), and mice in the control group were injected with normal saline. One week later, 2.5% (g/mL) DSS (Yeasen Biotechnology, China) was added to the drinking water for 1 week, followed by 2 weeks of regular drinking water in the model group. The DSS treatment was repeated for one additional cycle. No intervention was given to the control group after injection with normal saline. All mice were sacrificed through cervical dislocation in the ninth week by inhaling ether. Colorectal tissues (from above the anus to the cecum) were cut out from each mouse and photographed after longitudinal anatomy. The colorectal tissues were inspected by two independent observers. Then, 4 cm length of colorectal tissues were excised, and embedded in 4% formaldehyde to be used for H&E staining and immunohistochemistry analysis.

**Xenograft mouse model**

For tumor growth assays, a total of 8 × 10^6^ SW620 cells stably transfected with lv-TRPC1 (TRPC1) or lv-Control (Vector), sh-TRPC1 or sh-Control in 0.2 mL RPMI1640 medium was independently injected subcutaneously into both dorsal flanks of male nude mice (4/group, 18 to 22 g, Male). The tumor volume was measured every 2 days and calculated by length × width^2^ × 0.5. No randomization was performed. Data was analysed from all mice included. All mice were sacrificed through cervical dislocation after 24 days by inhaling ether. After excising and weighing, tumors were photographed and kept for further analysis.

***In vivo* metastasis assay**

For establishment of *in vivo* lung metastasis model, 5 × 10^6^ SW620 cells stably overexpressing TRPC1 or the vector cells was injected into the tail vein of nude mice (4/group, 18 to 22 g, Male). No randomization was performed. Data was analysed from all mice included. After 30 days, all mice were sacrificed through cervical dislocation by inhaling ether to observe the tumor metastasis in their lungs. The lung tissues were inspected by two independent observers. The metastasis tissues were photographed and further analyzed by H&E and Ki67 staining.

Experiments on animals were performed in accordance with China animal welfare legislation and were approved by the relevant authorities (Approval Form of IEC, First Affiliated Hospitalof Fourth Military Medical University).

**Western blot analysis**

Western blotting was performed using a SDS-PAGE electrophoresis system as previously described^1^. Signals were detected using a chemiluminescence apparatus (Tinon Biotech, China). Protein bands were analyzed by the ImageJ software. Anti-TRPC1 (Cat#sc-133076, Santa Cruz), anti-Calmodulin (Cat#BS9898M, Bioworld), anti-PI3K (Cat#60225-1-lg, Proteintech), anti-phospho PI3K p85α (Cat#AF3242, Affinity), anti-AKT (Cat#10176-2-AP, Proteintech), anti-phospho AKT ser473 (Cat#4060, Cell Signaling Technology), anti-CDK1 (Cat#19532-1-AP, Proteintech), anti-CyclinB1 (Cat#55004-1-AP, Proteintech), anti-N-cadherin (Cat#66219-1-lg, Proteintech), anti-E-cadherin (Cat#60335-1-lg, Proteintech), anti-Snail1 (Cat#13099-1-AP, Proteintech), anti-Slug (Cat#21129-1-AP, Proteintech), anti-β-actin (Cat#20536-1-AP, Proteintech) and anti-LC3 (Cat#14600-1-AP, Proteintech) were used as primary antibodies. Results were expressed as the ratio relative to β-actin.

**Co-immunoprecipitation (co-IP) assay**

The SW620 cells stably transfected with lv-TRPC1 (TRPC1) or lv-Control (Vector), sh-TRPC1 or sh-Control were harvested and pretreated by 80 µL control Agarose resin (Thermo Fisher Scientific, USA) for 1 h at 4℃. 10ug anti-PI3K (Cat#60225-1-lg, Proteintech) or IgG (Cat#B00050, Bioworld) antibody combined with 20 µL Protein A/G Plus Ararose (Thermo Fisher Scientific, USA) were mingled in centrifugal column for 1 h at room temperature. Subsequently, pretreated supernatant were added to the centrifuge column containing specific antibody-crosslinked resin and incubated at 4℃ overnight with gentle rotation. After extensive washing, proteins were eluted with low-pH buffer and separated by SDS-PAGE gel, followed by western blot analysis with indicated antibodies as described above.

**Immunofluorescence staining**

Immunofluorescence analysis was performed as previously described^2^. Briefly, cells or tissues were blocked with 5% bovine serum albumin (BSA) for 90 min, then incubated with specific primary antibodies overnight at 4℃. After that, fluorescence-conjugated secondary antibodies were incubated at room temperature for 70 min. After a rinse, the cells or tissues were stained with DAPI (Leagene Biotech, China) for 10 min. Anti-Calmodulin (Cat#BS9898M, Bioworld), anti-TRPC1 (Cat#sc-133076, Santa Cruz), and anti-PI3K (Cat#BS91073, Bioworld) antibodies were used as primary antibodies. FITC-IgG (Cat#SA00003-1, Proteintech), AMCA-IgG (Cat#SA00010-1, Proteintech), Cy3-IgG (Cat#SA00009-2, Proteintech), and Alexa Fluor 467 (Cat#A0473, Beyotime) antibodies were used as secondary antibodies. Images were observed under a confocal microscope (FV1000, Olympus, JPN), using 400× or 600× magnification.

**Immunohistochemistry**

IHC staining for tissue sections were done as described previously^3^. In brief, 4 μm paraffin colorectum slices were blocked in 5% BSA for 60 min. Next, the tissues were incubated with specific primary antibodies overnight at 4℃, and then incubated with corresponding secondary antibodies at room temperature for 70 min. Anti-TRPC1 (Cat#sc-133076, Santa Cruz) and anti-Ki67 (Cat#27309-1-AP, Proteintech) were used as primary antibodies. Data were collected from an average of 5 randomly selected areas in a random section from 3 examined tissues. Results were analyzed using Image Pro Plus 6.0 software (Media Cybernetics, Inc., USA) and expressed as the mean optical density.

**Cell viability assays**

Cell viability was determined by MTT assays. Briefly, CRC cells were seeded in 96-well plates at 5×10^3^ cells/well. After incubation for 1 to 8 days, the medium was replaced with MTT solution and the absorbance of each well was measured at 570 nm through microplate reader. Relative cell viability = number of cells on each day / number of cells on first day.

**Plate colony formation assays**

Plate colony formation assay was performed as previously described^4^. Briefly, cells were inoculated in 6-well plates at a density of 1000 cells per well. After cells were stabilized, the culture medium containing LY294002 (10 μM, MedChemExpress, USA) or not was replaced every 2 days for 14 days. Colony formation was photographed and evaluated after fixing the cells with 4% paraformaldehyde and staining with 0.5% crystal violet.

**Cell cycle assays**

Cells were trypsinized, washed with cold phosphate-buffered saline (PBS) and fixed in 70% ethanol 3 h at 4℃. Cells were then washed twice with PBS, and stained with PI at room temperature for 1 h. Cell cycle distribution was measured by the Becton-Dickinson FACScan System (Franklin Lakes, NJ, USA).

**Cell migration and invasion assays**

Cell migration and invasion assays were performed with trans-well chambers as described in previous study^1^. For cell migration assays, cells were detached and washed with PBS, and a total of 5×10^4^ cells in serum-free medium was added to the upper chamber (24-well insert; pore size, 8 μm; Corning, NY). Medium with 20% FBS was added to the bottom wells of the chambers. The cells that had migrated to the lower face of the filters were fixed with 4% paraformaldehyde, stained with crystal violet solution, photographed under microscope and quantified. Similar inserts coated with matrigel were used to determine the invasive potential in the invasion assay. For LY294002 treatment group, cells were preprocessed with LY294002 (10 μM) for 48 h before adding to the upper chamber. Relative cell migration/invasion ability = absorbance of indicated group / absorbance of control.

**Analysis of Ca^2+^ levels**

TRPC1 silenced and controlled cells (HT29, SW620, HCT116) were plated at about 1.0 × 10^4^ cells on 10-mm glass coverslips. After 24 h, cells were loaded with 5 μm Fura-2/AM for 25 min at 37 ℃, No calcium solution (contains EGTA7.6mg and 1g glucose) was perfused for 3 minutes, and placed on the stage of an inverted microscope. After adding thapsigargin (TG), intracellular calcium was consumed. Then, cells were given buffer with an extracellular Ca^2+^ of 2 mM to assay for TRPC1 channel mediated Ca^2+^ entry. The emitting fluorescence (F340 and F380) were recorded by a digital wide-field fluorescence imaging system (TILL Photonics GmbH, Gräfelfing, Germany). Ratio-images were calculated from F340 and F380 pictures after background correction. Single cells were marked and F340/F380 was plotted versus time.

**Supplementary references**

1. Li Y*, et al*. Paris saponin VII inhibits growth of colorectal cancer cells through Ras signaling pathway. *Biochem. Pharmacol.* 2014; **88**: 150-157.

2. Sun Y*, et al*. Apple polysaccharide prevents from colitis-associated carcinogenesis through regulating macrophage polarization. *Int. J. Biol. Macromol.* 2020; **161**: 704-711.

3. Sun Y*, et al*. Modified apple polysaccharide influences MUC-1 expression to prevent ICR mice from colitis-associated carcinogenesis. *Int. J. Biol. Macromol.* 2018; **120**: 1387-1395.

4. Zhou H*, et al*. Paris saponin VII extracted from trillium tschonoskii suppresses proliferation and induces apoptosis of human colorectal cancer cells. *J. Ethnopharmacol.* 2019; **239**: 111903.

**Supplementary Figures and Legends**

**Supplementary Fig. S1: Correlation between TRPCS mRNA levels and survival and mutation in CRC patients. A** Cbioportal database analysis TRPC1 expression in function of PI3K, BRAF, K-RAS mutational status. **B** Kaplan-Meier survival analysis of disease-free survival according to the *TRPC1* mRNA expression in the TCGA dataset. **C** Kaplan-Meier survival analysis compared CRC patients with high or low *TRPC3*, *TRPC4*, or *TRPC6* mRNA expressions in the TCGA dataset. **, *P* < 0.01 *vs.* WT group. ^NS^, *P* > 0.05 *vs.* WT group.

**Supplementary Fig. S2: Successful silence of TRPC1 and CaM, and successful overexpression of TRPC1 were identified.** **A** Representative fluorescence (Flu) imaging of the CRC cell lines (HCT116, HT29, and SW620) with TRPC1 silencing or overexpression. The transfected lentiviral vector carried green fluorescence. **B** The levels of TRPC1 were examined by western blot analysis (n = 3). β-actin was used as an internal control. *, *P* < 0.05; **, *P* < 0.01 *vs.* control. **C** The successful silence of CaM was verified in the Lv-Con and Lv-TRPC1 SW620 cells by western blot analysis (n = 4). **, *P* < 0.01 *vs.* Lv-Con group. ^##^, *P* < 0.01 *vs.* Lv-TRPC1 group.

**Supplementary Fig. S3: TRPC1 protein expression in AOM/DSS-induced CRC mice model.** **A** Scheme for the AOM/DSS-induced CRC mice model. Sac, sacrifice. **B** Protein expressions of TRPC1 in the colorectal tissues from saline or AOM/DSS-induced mice were assessed by IHC. The representative tissues are shown in the left panel along with the quantitative analysis of TRPC1 integrated optical density (IOD) in the right panel (n = 5). **, *P* < 0.01 *vs.* Control-WT group. ^##^, *P* < 0.01 *vs.* AOM/DSS-WT group. **C** Ki67 staining of the metastatic lung tumor tissues from nude mice inoculated with SW620 cells stably overexpressing TRPC1 or vector. The representative tissues are shown in the upper panel along with the quantitative analysis of Ki67 in the lower panel (n = 4). **, *P* < 0.01 *vs.* vector.

**Supplementary Fig. S4: TRPC1 promotes cell invasion and migration *in vitro*.** **A** TRPC1 knockdown inhibited the invasive and migratory capabilities of HCT116 cells as measured by the transwell assay. Typical results are shown in the upper panel along with the statistical analysis in the lower panel (n = 5). **B** Overexpression of TRPC1 facilitated the invasive and migratory capabilities of HT29 cells as measured by the transwell assay (n = 5). **, *P* < 0.01 *vs.* control.

**Supplementary Fig. S5: Western blotting quantification. A and C,** The relative protein levels of EMT regulators, including E-cadherin, N-cadherin, Snail1, and Slug, detected in the SW620 cells over-expressing *TRPC1* and SW620 cells with stably suppressed *TRPC1* expression by western blot analysis (n = 3) **. B,** Knockdown of endogenous *TRPC1* in SW620 cells reduced the protein levels of CyclinB1 and CDK1 (n = 3). *, *P* < 0.05; **, *P* < 0.01 *vs.* control. **D,** The levels of CaM, PI3K, phosphorylated-PI3K (p-PI3K p85), AKT, and p-AKT were examined by western blot analysis in the SW620 cells with silenced or enhanced *TRPC1* (n = 3). *, *P* < 0.05; **, *P* < 0.01 *vs.* shControl group. ^#^, *P* < 0.05; ^##^, *P* < 0.01 *vs.* vector group. **E,** Effects of LY294002 on AKT activation in the SW620 cells with TRPC1 overexpression or vector by western blot (n = 3). *, *P* < 0.05 *vs.* Lv-Con group. ^##^,*P* < 0.01 *vs.* Lv-TRPC1.

**Supplementary Fig. S6: LY294002 affects cell invasion, migration and autophagy of the SW620 and HT29 cells induced by TRPC1 overexpression.** **A, B** PI3K inhibitor restrained the invasive and migratory capabilities of the TRPC1 overexpressed HT29 and SW620 cells as shown by the transwell assay. Typical results are shown in the upper panel along with the statistical analysis in the lower panel (n = 5). **C,** The relative protein levels of EMT regulators, including E-cadherin, N-cadherin, Snail1, and Slug, detected in the SW620 cells over-expressing *TRPC1*. **D,** The relative protein levels of autophagy regulators in the SW620 cells over-expressing *TRPC1*. **E,**  Co-IP data on normal colonic epithelial NCM460 cells.*, *P* < 0.05; **, *P* < 0.01 *vs.* Lv-Con group. ^##^, *P* < 0.01 *vs.* Lv-TRPC1 group.

**Supplementary Fig. S7: The effects of CaM in TRPC1-induced CRC progression. A-B,** Effects of CaM silencing on PI3K and AKT activation in the SW620 cells over-expressing *TRPC1* and SW620 cells with stably suppressed *TRPC1* expression by western blot analysis (n = 3) *, *P* < 0.05; **, *P* < 0.01 *vs.* Lv-Con group. ##, *P* < 0.01 *vs.* Lv-TRPC1 group. **C,** Silence of CaM suppressed colony formation in the SW620 cells silence TRPC1 (n = 5). **D,** Silencing CaM restrained the migratory capabilities of the TRPC1 silenced SW620 cells as determined by the transwell migration assay (n = 5). **, *P* < 0.01 *vs.* sh-Con+siCon group. . **E,** Cbioportal database analyzed the overall survival for CaM expression in patients stratified according to TRPC1 mRNA expression in both PI3K mutated and unmutated subsets.

**Supplementary Fig. S8:** **Analysis of Ca2+ levels and TRPC1 expression. A-C,** Single cells were marked and F340/F380 was plotted versus time. **, *P* < 0.01 *vs.* shControl group. ^NS^, *P* > 0.05 *vs.* shControl group. **D,** The confocal immunofluorescence assay showed that TRPC1 was expressed in the cytoplasm and cytomembrane of colorectal cancer cell lines HT29, SW620 and HCT116.

**Supplementary Fig. S9: A TRPC1/CaM/PI3K ternary complex model built from three PDB structures.** TRPC1 (homologous modeling according to TRPC4 protein), CaM, and PI3K were used as target proteins. The PDB codes are 7b0j (TRPC4), 2LL6 (CaM), and 4ovv (PI3K). We predicted models for the binary interactions between TRPC1 and CaM, and CaM and PI3K based on available literature data.
